# Predicting sustainability performance in construction projects using machine learning: a comparative study

**DOI:** 10.1038/s41598-025-32658-8

**Published:** 2026-01-02

**Authors:** Ahmed Ali A. Shohan, Mohammed Alshayeb, Saleh Alsulamy

**Affiliations:** 1 Architecture Department, College of Architecture & Planning, King Khalid University, Abha, 61421 Saudi Arabia; 2 Architecture Department, College of Architecture and Planning, King Khalid University, Abha, Saudi Arabia

**Keywords:** Carbon emissions prediction, Machine learning, Sustainability assessment, SHAP interpretation, Energy science and technology, Engineering, Environmental sciences, Environmental social sciences, Mathematics and computing

## Abstract

The construction sector plays a major role in global environmental degradation, contributing significantly to carbon emissions, energy consumption, and waste generation. Despite this urgency, limited studies have explored predictive modelling of sustainability performance using survey-based project data, particularly within Saudi Arabia. This study addresses this gap by applying supervised machine learning techniques to predict carbon emissions and classify projects into emission-level categories. A structured survey generated 150 validated responses from key stakeholders across major Saudi cities, covering 19 project and sustainability attributes. Three machine learning models, Support Vector Machine (SVM), Random Forest (RF), and Extreme Gradient Boosting (XGB) were trained and evaluated using nested 10 × 5-fold cross-validation. RF achieved the strongest regression performance (mean CV R^2^ = 0.439 ± 0.247; test R^2^ = 0.734) and the highest classification accuracy (0.790 ± 0.094 CV; 78% test), outperforming SVM and XGB. SHAP analysis consistently identified waste generation, energy consumption, and project duration as the most influential predictors of carbon emissions. The findings deliver a data-driven framework for early sustainability assessment and support informed policy and planning aligned with Saudi Vision 2030.

## Introduction

 Sustainability is increasingly becoming a top priority in the construction sector owing to the fact that it carries a huge environmental impact. The construction sector consumes approximately 35% of global energy, emits 40% of CO_2_ emissions, and generates approximately one third of solid waste generation^1^. All these effects have a major role in causing climate change and the depletion of resources, and hence measuring and enhancing sustainability performance in construction projects is absolutely essential^1,2^. The most critical environmental performance measures carbon emissions, waste generation, and energy consumption are comprehensively used to measure the construction process’s sustainability^2^. Enhancing these parameters is crucial for the sector to achieve climate goals and sustainable development goals.

Carbon emissions, waste, and energy consumption have become significant sustainability indicators in the construction industry. Carbon emissions linked to the construction practice come from the burning of fuel in the machinery, electricity used on site, and natural carbon in the materials, thus directly contributing to climate change^2^. Construction and demolition waste, responsible for about 30–40% of solid waste in landfill, is indicative of inefficient use of materials and causes significant environmental harm^3,4^. Greater energy consumption in construction activities, diesel for machinery and electricity for site consumption adds to costs as well as increases greenhouse gas emissions. Therefore, green building practice and assessment systems especially address the reduction of energy consumption, carbon emissions, and waste generation, thus making a focus on the three factors to undertake sustainable construction practice^5,6^.

Early-stage measurement and forecasting of sustainability performance can make a significant difference in results. Traditional measurement methods, such as life-cycle analysis, are inclined to require detailed data and are applied late in the project timeline, limiting their potential to inform forward-looking decisions^7,8^. This has created a lot of interest in predictive and data-driven approaches. Specifically, machine learning (ML) techniques have demonstrated a lot of promise for predicting the sustainability performance of construction projects by analysing project-specific data to estimate key environmental metrics such as carbon emissions, energy use, and waste generation^9–11^. Research suggests that ML models can significantly improve the accuracy of construction waste prediction and enable enhanced recycling and resource planning strategies^12^. The use of historical data by ML models has the potential for making initial quick estimates of environmental impacts, thus enabling project teams with decision-making data to prevent adverse effects.

Despite this progress, notable research gaps remain. Many prior studies focus exclusively on predicting a single sustainability metric, such as waste generation or energy consumption, while fewer address carbon emissions specifically and even fewer attempt to classify projects based on emission intensity^9,10,13,14^. Furthermore, a significant portion of the existing literature relies on simulated data or narrowly defined case studies, limiting the generalizability and practical application of the models developed. This issue is especially pronounced in the Middle East, where the construction industry plays a major role in national emissions. In Saudi Arabia, for example, the construction sector is estimated to contribute nearly 38% of the country’s total carbon emissions. Despite the strategic emphasis on environmental sustainability under the Vision 2030 initiative, there remains a lack of empirical studies that apply machine learning to predict and classify carbon emissions using survey-derived estimates from Saudi construction projects^15^.

To address these gaps, this study proposes a comprehensive machine learning framework for predicting and classifying carbon emissions during the construction phase using survey-based project data from Saudi Arabia. By applying supervised learning techniques and model interpretation tools, this research aims to improve sustainability assessment and support data-driven decision-making in line with Saudi Vision 2030. The key contributions of this study are as follows:

- Development of a dual-purpose machine learning model that supports both regression and classification of carbon emissions.
- Use of a unique, stakeholder-informed dataset collected from 150 construction projects across major Saudi cities.
- Comparative analysis of three established ML algorithms namely Support Vector Machine (SVM), Random Forest (RF), and Extreme Gradient Boost (XGB) for sustainability prediction in construction.
- Integration of SHAP (SHapley Additive exPlanations) to identify and interpret key drivers of carbon emissions.
- Actionable policy recommendations to promote low-carbon construction practices in the Saudi context.

This study provides an actionable, data-driven framework that can help project managers, contractors, and policymakers make informed decisions to reduce the environmental impact of construction projects in line with sustainable development objectives.

## Literature review and research significance

Sustainable construction has become a major research focus as the building industry significantly contributes to global greenhouse gas emissions. Approximately 28% of worldwide CO_2_ emissions are attributed to construction activities^16^​. Reducing this footprint during the construction phase is critical for meeting climate targets. Traditionally, detailed life-cycle assessment (LCA) methods are used to estimate a project’s carbon emissions, but these can be time-consuming and data-intensive^16^. Recent advances in ML offer a scalable alternative to traditional assessment methods by learning complex patterns from historical data and enabling early-stage prediction of key sustainability metrics. This aligns with the construction industry’s shift toward digital transformation and low-carbon practices^17–23^​.

### Application of ML in construction sustainability

A number of studies have leveraged ML to predict carbon emissions associated with construction processes and building projects. Traditional estimation methods (e.g. linear regression or manual calculation) often struggle with the nonlinear relationships between design/construction parameters and resulting CO_2_ emissions^19,20,24,25^. ML methods can better capture these relationships. For instance, Fang et al.^26^ developed a RF model to predict construction-stage carbon emissions based on early design information from 38 building projects, achieving an R^2^ of approximately 0.64​. While moderate, this accuracy was an improvement over simpler models and demonstrated the viability of using ensemble decision trees for carbon estimation in the pre-construction phase. More recent work has pushed prediction accuracy much higher. Razi and Ansari^27^ tested various ML algorithms to forecast CO_2_ emissions of construction schedules under different plan scenarios; their best model, an artificial neural network (ANN), achieved a CO_2_ prediction accuracy of about 98% (R^2^ ≈ 0.98) on test data​. This study integrated ML predictions into a multi-objective optimization, balancing time, cost, energy usage, and carbon emissions, to support sustainable project planning​. Similarly, Hou et al.^28^ proposed an ensemble approach combining Bayesian hyperparameter optimization with XGB to model carbon emissions in China’s construction sector. The optimized XGB model delivered high predictive performance with R^2^ around 0.91, substantially outperforming a baseline RF model on the same data​​.

Another critical aspect of sustainability is minimizing construction and demolition waste. Accurate prediction of waste generation can facilitate better waste management plans, higher recycling rates, and lower landfill usage. Researchers have increasingly applied ML to forecast waste quantities and composition for construction projects. Support vector machines have shown promising results in this domain. Hu et al.^19^ developed an SVM-based model to estimate construction waste generation for commercial building projects, using improved on-site measurement data as inputs. Their SVM model achieved an R^2^ of ~ 0.87, significantly outperforming a back-propagation neural network (R^2^ ~ 0.75) and a multiple linear regression baseline (R^2^ ~ 0.62) on the same dataset​. This indicates that SVM’s ability to model nonlinear relations gave it an edge in predicting waste volumes. However, ML models may face challenges when training data are limited – a common situation for project-specific waste data. To address this, Cha et al.^29^ experimented with enhancing SVM predictions through feature extraction techniques. By applying a categorical principal components analysis to a small demolition waste dataset, they improved an SVM regression model’s performance from essentially no predictive power (R^2^ ≈ 0) to about R^2^ = 0.59​. In parallel, tree-based ensemble models have been effectively used for waste prediction. Cha et al.^25^ also reported that a RF model could predict building demolition waste with R^2^ in the range of 0.55–0.80 despite the small training sample, thanks to RF’s robustness against overfitting and ability to handle heterogeneous inputs​. Notably, in that study gradient boosting performed on par with (and in some cases slightly better than) RF, highlighting that boosting algorithms like XGB can attain robust results even with limited data and categorical variables​. Beyond conventional ML, deep learning approaches have been applied when larger integrated datasets are available. For example, Akanbi et al.^23^ applied deep neural networks to predict the quantities of recoverable materials from building demolition. Their BIM-driven deep learning model achieved an average R^2^ of 0.97 in estimating total salvageable waste, with performance as high as R^2^ ~ 0.99 for certain material categories​. Such high accuracy suggests that, given comprehensive project data, advanced ML can very precisely foresee waste generation, enabling near-zero-waste construction goals. Recent studies from developing-country contexts have likewise adopted ensemble ML for C&D-waste forecasting under data-scarce conditions. For example, an optimized XGB model achieved strong accuracy using limited datasets in Egypt/China and was proposed specifically for developing settings^17^. A separate case study from Tehran by Jafari et al.^30^ showed that AI/ML models (SVM, ANFIS, etc.) reliably predict monthly C&D waste where official records are sparse. Recent reviews also highlight the growing use and competitive performance of boosted trees and other ensembles for C&D-waste estimation and management^11,31^. In summary, the application of ML to construction waste prediction has shown clear benefits, from support vector machines to ensemble and deep learning models, a wide range of ML techniques have demonstrated reliable performance in predicting sustainability indicators, particularly when models are tuned or enhanced with feature engineering, all contributing to more sustainable waste management practices on construction sites.

Moreover, energy use during construction (e.g. fuel for machinery, electricity on-site) and the long-term energy performance of buildings are integral sustainability considerations. ML techniques that accurately predict energy consumption can support both efficient construction practices and the design of energy-efficient structures. Within the built environment domain, numerous studies have demonstrated that data-driven models outperform physics-based or simpler statistical models in energy consumption forecasting^32–34^. A recent review of building energy prediction by Olu-Ajayi et al.^33^ found that algorithms like SVM, RF, and ANN consistently yielded lower error and higher accuracy than traditional regression across a majority of cases​. These models learn from historical energy usage patterns, weather, and operational data, enabling more reliable predictions of future energy demand. For instance, SVMs have been used to forecast building HVAC energy loads with greater precision than linear methods, especially when ample sensor data are available​^33^. In the context of construction projects, ML can be applied to optimize equipment operation schedules and site logistics to reduce energy waste. Razi and Ansari’s multi-factor model is one example, where an ANN was used not only for carbon predictions but also to estimate energy consumption of project activities, aiding in optimizing the trade-offs between time, cost, carbon, and energy use^27^​. By integrating such ML-driven energy estimates, project managers can identify scenarios that minimize fuel and electricity usage on site (e.g. by adjusting work sequences or machinery deployment) without compromising project timelines. Although dedicated research on ML for construction-phase energy consumption is still emerging, lessons from building operation models and multi-objective studies indicate a strong potential for SVM, RF, and XGB to contribute to energy-saving strategies during construction.

However, despite growing global research on ML-driven sustainability assessment, there remain important gaps and regional disparities. Much of the existing literature originates from North America, Europe, and East Asia, whereas applications in the Middle East (and specifically Saudi Arabia) are relatively sparse. Saudi Arabia’s construction sector is enormous and rapidly expanding under initiatives like Vision 2030, and it faces pressing sustainability challenges such as the construction industry in Saudi alone accounts for roughly 38% of national CO_2_ emissions^15^​. This underscores an urgent need for advanced tools to evaluate and curb emissions in Saudi construction projects. Yet, few studies to date have examined project-level carbon footprint prediction in the Saudi context using ML approaches. Most ML models in literature are developed on datasets from other regions, which may not directly capture the practices, materials, and climate conditions unique to Saudi construction projects. Additionally, prior studies often focus on a single aspect (e.g. predicting emissions or waste separately) and may not be integrated into decision-making processes on active projects.

While numerous worldwide studies confirm that ML techniques (SVM, RF, XGB, etc.) can successfully predict carbon emissions and other sustainability indicators for construction projects, these advances have not been fully translated to all contexts. There is a clear gap in the application of these models to the construction stage of projects in Saudi Arabia and similar regions, where localized data and validation are lacking. Moreover, existing research has largely been exploratory demonstrating predictive performance but there remains a need to integrate ML predictions with multi-criteria decision-making (MCDM) frameworks to guide project stakeholders in real time. This research aims to fill these gaps by developing a robust ML-based model (using state-of-the-art algorithms like SVM, RF and XGB) tailored to predict construction-stage carbon emissions under Saudi Arabian project conditions. By combining this predictive model with a sustainability assessment framework, the study will extend the current body of knowledge and provide a practical tool for engineers and policymakers to reduce carbon footprints during construction. This contribution is significant as it addresses a regional gap in literature and links predictive analytics with actionable sustainability strategies, moving the construction industry closer to its low-carbon goals.

### Theoretical framework and hypotheses

The study is grounded in technology-adoption perspectives that explain when decision-makers in construction perceive ML-based decision support as credible and usable. The Technology Acceptance Model (TAM) and UTAUT emphasise perceived usefulness and ease of use as key determinants of adoption in professional settings, offering a lens for interpreting how predictive tools become acceptable inputs to sustainability assessments in projects^35,36^. In the project delivery context, these constructs help connect model accuracy and transparency to stakeholder willingness to integrate predictions into routine planning and reporting.

Complementing adoption theory, sustainability outcomes are situated within the Triple Bottom Line (TBL), linking environmental (CO_2_, energy, waste), economic (budget, efficiency), and social considerations (safety, community) so that model outputs are interpreted beyond purely technical fit^37^. The Diffusion of Innovations perspective further clarifies how tools spread in the sector when they demonstrate relative advantage, compatibility with existing workflows, and observable results^38^. Together, these lenses motivate the emphasis on models that balance generalisation with explainability for practitioner uptake, directly informing the hypotheses^39–41^ as stated below.

- **H1**: Tree-based ensembles (RF, XGB) achieve higher predictive accuracy than kernel SVM on mixed, tabular project data with nonlinear interactions.
- **H2**: SHAP-based interpretation highlights project-level drivers (e.g., material mix, project size, duration, waste) as significant contributors to construction-phase CO_2_.
- **H3**: In context prioritizing early, actionable assessment, boosting-based models (XGB) provide superior generalization relative to bagging (RF) and SVM due to built-in regularization and interaction learning.
- **H4**: Demonstrable accuracy and explainability increase the likelihood of ML integration into sustainability workflows in construction organizations.

## Research methodology

### Questionnaire development

To effectively capture the factors influencing sustainability performance during the construction phase, a structured questionnaire was meticulously developed based on a comprehensive review of existing literature, validation by industry experts, and a small-scale pilot test. The aim was to gather reliable and context-specific data on key environmental performance indicators namely carbon emissions, energy consumption, and construction waste generation while also considering social and economic dimensions of sustainability. The finalized questionnaire was subsequently circulated among construction firms in major Saudi cities including Jeddah, Makkah, Riyadh, Abha, Dammam, and Madinah.

#### Indicator identification through literature review

The initial step in developing the questionnaire involved an extensive literature review to identify relevant sustainability indicators and their use in prior research. Existing research on green construction, its assessment tools (LEED and BREEAM), and indicator-based systems served as the basis for preparing the item selection. Important environmental aspects such as carbon footprint, energy use intensity, and solid waste generation were identified repeatedly as key indicators of sustainability performance at the project level within the construction industry^42–44^.

Social and economic indicators were also taken from other documents concentrating on community perception, worker safety, project costs, and return on investment (ROI)^45,46^. These factors were used to create Likert-scale sections of the questionnaire designed for the respondents to articulate the level of perceived importance towards each factor for the sustainable project execution. This is in line with the approaches used in other similar sustainability assessments within the construction industry^47^.

#### Expert review and contextual adaptation

Following the creation of the first draft of the questionnaire, a construction industry panel comprising five experts, including a senior academic researcher and a sustainability consultant, was convened to evaluate its relevance to tracking construction industry standards in Saudi Arabia as well as its content validity. The construction industry in Saudi Arabia was proficiently aligned with the common terms used in several items owing to previously be received feedback. For instance, project budget ranges and experience were adapted to local standards and regulatory terminology.

The objective of this validation was to ensure that the instrument is as complete as possible, with respect to all international standards for measurements of sustainability, and at the same time, practical and useful for local professionals. Other studies have also adopted similar validation techniques to improve the reliability of the developed questionnaires for the specific sectors^48,49^.

#### Survey instrument development and validation

The survey instrument was grounded in the triple-bottom-line view of sustainability and operationalized from established indicator families in the construction literature and assessment frameworks. Environmental items emphasized carbon emissions, energy consumption, and construction and demolition waste; economic items focused on initial cost, lifecycle cost, and return on investment; and social items addressed health and safety and community impact. Item wording and coverage were adapted from prior work and indicator lists in green construction and materials selection^42–46^, with phrasing tailored to the construction phase and Saudi project context. Importance judgements were captured on a 5-point Likert scale (1 = Not important to 5 = Very important), while project-level outcomes (carbon emissions, energy use, waste) were collected as numerical values for modelling.

**Fig. 1:**
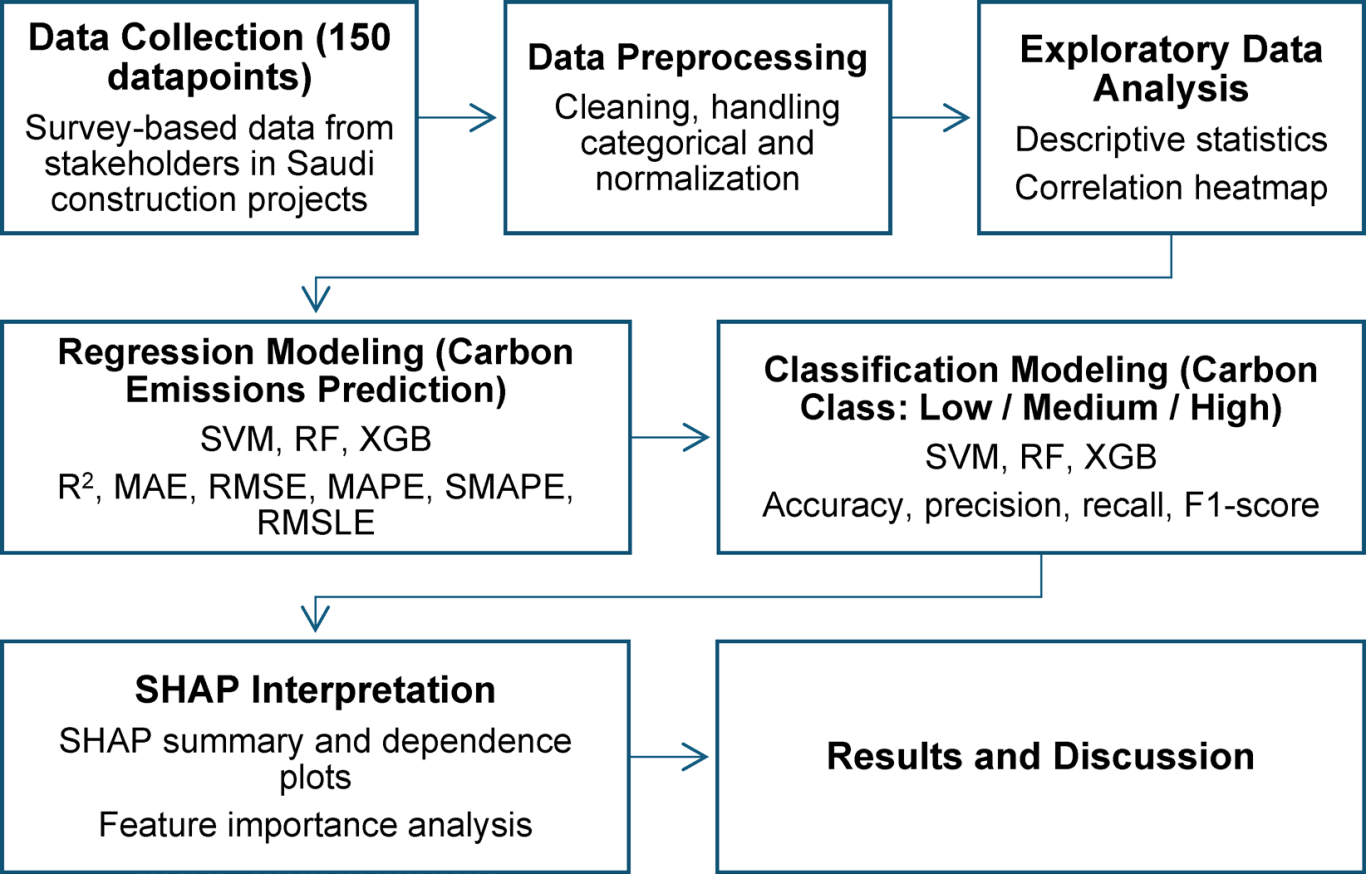
Pearson multicollinearity heat map.

To ensure content validity beyond expert review, study implemented a quantitative Content Validity Index (CVI) procedure: five domain experts independently rated each item’s relevance on a 4-point scale; item-level CVIs (I-CVIs) and a scale-level CVI (S-CVI/Ave) were computed, and items with low relevance were revised or removed. Cognitive pre-testing with pilot respondents (think-aloud and probing) was then used to refine terminology, ordering, and response options; local practice was reflected by aligning budget ranges and experience bands. Redundancy was screened during analysis via correlation diagnostics (Fig. 1), ensuring that retained items contributed distinct information to the subsequent machine-learning models. For completeness, constructs and example stems were also cross-checked against sectoral studies in the Gulf region to maintain contextual fit^45^ and related adoption/practice literature^46^.

#### Pilot testing

To assess clarity, response time, and respondent burden, a pilot version of the questionnaire was administered to a small sample of 10 professionals from various construction-related disciplines, including engineers, site supervisors, and environmental officers. The pilot test confirmed the feasibility of the instrument, and only minor adjustments were made to item sequencing and layout to improve user experience.

Pilot testing is a widely accepted strategy in construction research to reduce ambiguity, test response options, and optimize questionnaire length and flow^50^. This stage ensured that the final instrument could be distributed efficiently without compromising data quality.

#### Final questionnaire structure

The final questionnaire consisted of four main sections: (1) demographic and project profile information (e.g., role in project, years of experience, project size, material type), (2) importance ratings of sustainability indicators using a 5-point Likert scale, (3) actual values for project-specific outcomes (carbon emissions, energy consumption, waste generation), and (4) open-ended items for qualitative feedback.

The structured format enabled the collection of both subjective perceptions and objective performance data, which were later used as inputs and targets for machine learning models. The combination of categorical, Likert-scale, and numerical data supported a multi-dimensional analysis of sustainability performance in construction.

#### Sample selection and justification

The target population comprised key stakeholders involved in the construction phase, contractors, consultants, and clients working on projects in major Saudi cities (Jeddah, Makkah, Riyadh, Abha, Dammam, and Madinah). Invitations were distributed to organizations active in these regions to obtain a sample reflecting diversity in roles, project types, sizes/budgets, and materials. After screening for completeness and internal consistency, 150 valid responses were retained for analysis.

The adequacy of this sample was assessed from both statistical and empirical perspectives. First, an a priori power analysis for multiple-regression-type effects (Cohen’s f^2^ = 0.15, α = 0.05, power = 0.80) with 19 predictors indicate a minimum required N of ≈ 153. The achieved sample of *N* = 150 is very close to this threshold, yielding an achieved power of approximately 0.80, and therefore provides adequate sensitivity to detect medium-sized effects, which are of primary practical interest in this study. Moreover, as is common in applied sustainability research, small effect detection is less critical than identifying strong, actionable predictors of emissions. Second, for the three-class classification task (Low/Medium/High emissions), the achieved sample yields reasonable per-class counts after stratification, supporting model training without severe class imbalance; any residual imbalance was handled during modelling.

Empirically, prior studies in construction sustainability and related prediction problems have reported reliable models with equal or smaller datasets, including early-stage carbon-emission prediction from 38 projects^26^ and demolition-waste prediction using small training samples with tree ensembles and SVMs^25,29^.

#### Ethics approval and consent to participate

This study involving human participants (survey respondents) was reviewed and approved by the Research Ethics Committee at King Khalid University (Approval No. ECM#2024–3195, dated 30/12/2024). All procedures were conducted in accordance with relevant guidelines and regulations, including the Declaration of Helsinki. Informed consent was obtained electronically from all participants prior to data collection. The official ethical approval letter is provided in the supplementary file.

### Utilization of advanced machine learning techniques

#### Data cleaning and preprocessing

**Fig. 2:**
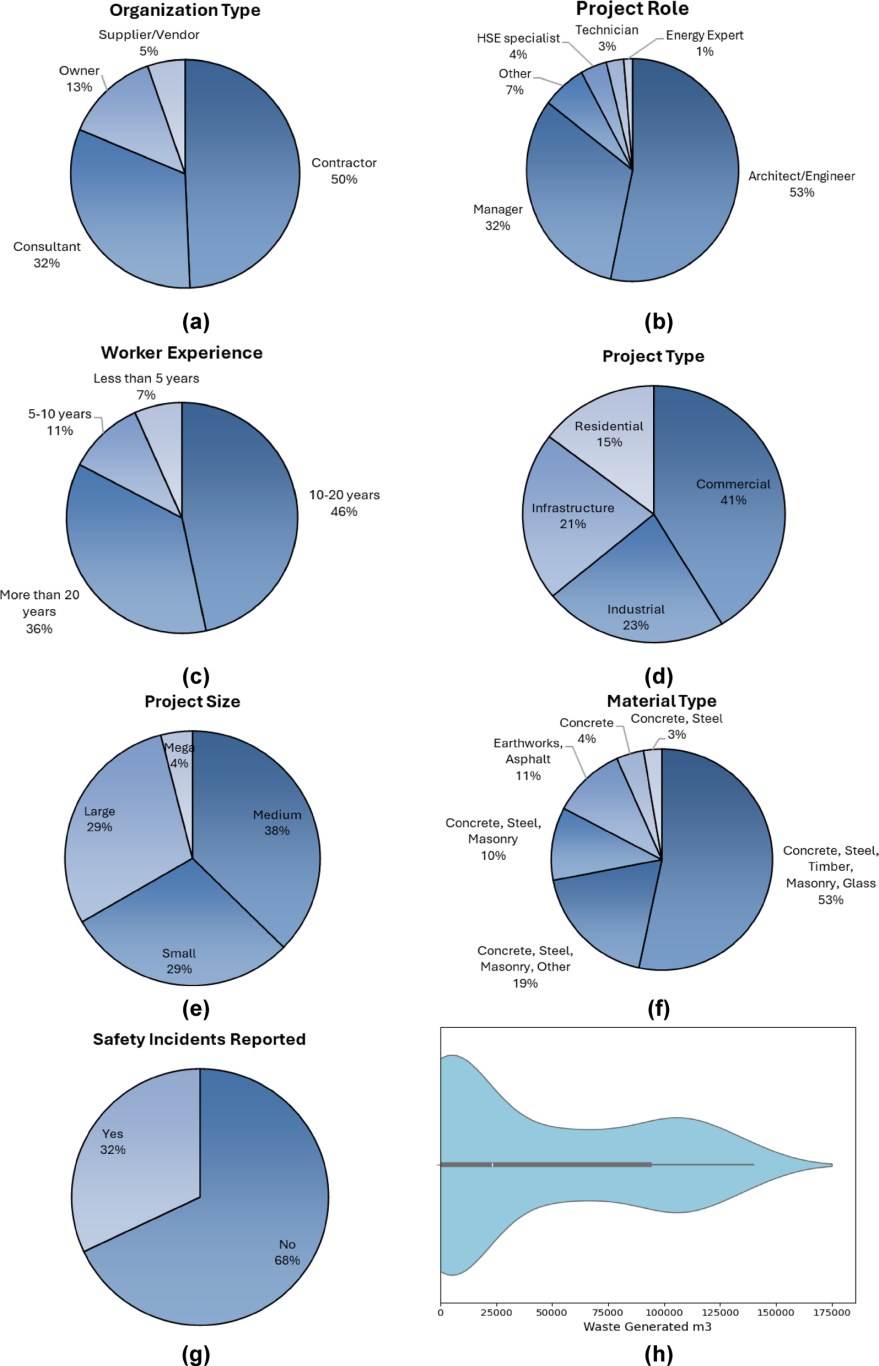

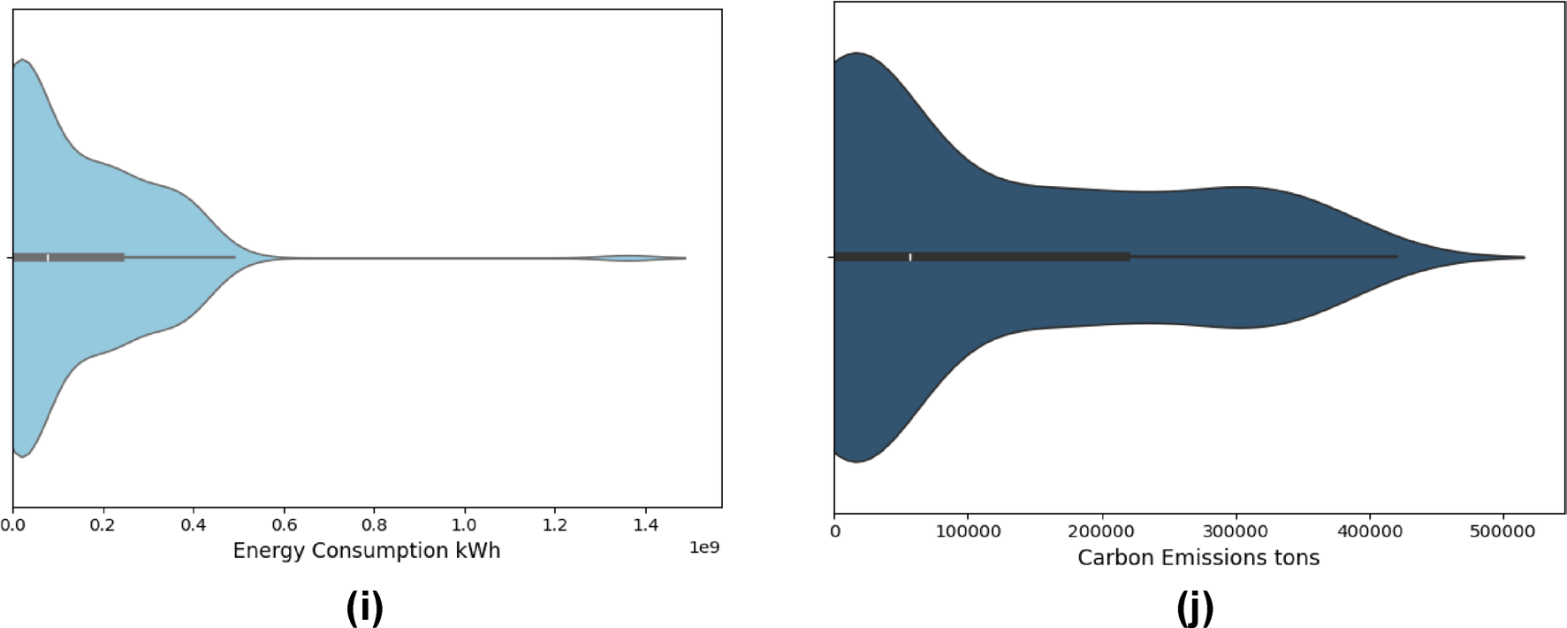
Structured methodology framework.

Prior to conducting any machine learning analysis, it is critical to ensure that the dataset is clean, consistent, and analytically robust. Data preprocessing is a fundamental step in preparing raw data for effective model training and evaluation^51–53^. In this study, the collected 150 questionnaire responses were carefully inspected to identify and correct errors, handle missing values, encode categorical variables, and standardize the input features where necessary. The structured flowchart of research methodology is illustrated in Fig. 2.

The initial phase involved screening the dataset for missing, inconsistent, or illogical responses. Entries with critical missing values in essential predictors, such as sustainability importance ratings or project demographic information, were either corrected where possible through participant follow-up or removed from the analysis. Missing values can introduce significant bias into machine learning models and affect generalizability^54^ ensuring complete data integrity was therefore prioritized.

Subsequently, categorical variables such as “Organization Type,” “Role in Project,” “Material Type,” and “Health and Safety Incidents” were encoded for analysis. For variables with nominal categories, one-hot encoding was applied to prevent artificial ordinality; this ensured that each stakeholder type or material category was represented distinctly without implying rank. For ordinal variables, such as Likert-scale sustainability ratings, the natural integer values were retained to preserve their inherent ordering^55,56^. Missing values were minimal (< 5% across all items) and were treated using simple imputation: mean substitution for continuous attributes (e.g., project size, cost) and mode substitution for categorical ones. To verify that this choice did not bias results, supplementary analyses with k-nearest-neighbor (KNN) imputation were conducted, producing similar outcomes. Continuous predictors were normalized differently depending on model requirements: for SVM, the StandardScaler (zero mean, unit variance) was applied, while for RF and XGB no scaling was performed since ensemble trees are invariant to feature ranges^57–59^. This approach is consistent with best practices in applied ML for sustainability datasets^44,46^. Additionally, while no log transformation of the target was performed, skewness in carbon-emission values was accounted for by evaluating percentage-based error measures such as MAPE, SMAPE, and RMSLE alongside RMSE.

#### Descriptive statistics and visualizations

To develop a deeper understanding of the 150 collected dataset prior to ML modeling, descriptive statistical analysis and visual exploration techniques were systematically employed. These methods provide a foundational step to identify data distributions, ensure data quality, and detect preliminary trends within the variables of interest^60,61^. To enhance readability in subsequent statistical tables, correlation matrices, and model visualizations, the dataset variables were abbreviated using concise notations (X1-X19). The dataset comprises 7 categorical variables and 12 numerical variables, as indicated in Table 1, which maps each input variable to its corresponding abbreviation.

**Table 1:** List of input variables with their corresponding abbreviations used in descriptive statistics, correlation analysis, and machine learning models.

Input Variables	Abbreviation	Input Variables	Abbreviation
**C**: Organization Type	X1	**N**: Importance of Waste Management	X11
**C**: Role in Project	X2	**N**: Importance of Indoor Environmental Quality (IEQ)	X12
**C**: Experience	X3	**N**: Importance of Initial Project Cost	X13
**C**: Project Type	X4	**N**: Importance of Lifecycle Cost	X14
**C**: Project Size/Budget	X5	**N**: Importance of Return on Investment (ROI)	X15
**N**: Project Duration (Months)	X6	**N**: Importance of Economic Efficiency	X16
**C**: Material Type	X7	**N**: Importance of Health and Safety standards	X17
**C**: Health and safety incidents reported	X8	**N**: Energy Consumption (kWh)	X18
**N**: Importance of Sustainable Materials	X9	**N**: Waste Generated (m^3^)	X19
**N**: Importance of Energy Consumption	X10		

*C = Categorical Variable, N = Numerical Variable.

**Fig. 3:**
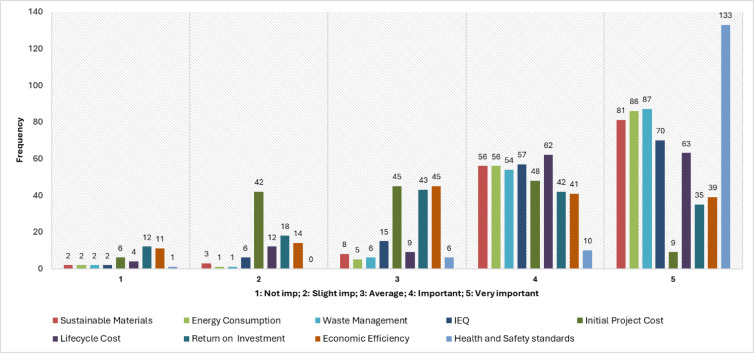
Descriptive Statistics (**a**) Organization Type, (**b**) Project Role, (**c**) Worker Experience, (**d**) Project Type, (**e**) Project Size, (f) Material Type, (**g**) Safety Incidents Reported, (**h**) Waste Generated, (**i**) Energy Consumption, (**j**) Carbon Emissions (Output).

Categorical variables such as Organization Type, Role in Project, Experience (Years), Project Type, Project Size/Budget, Material Type, and Health and Safety Incidents Reported were summarized using pie charts. These visualizations effectively illustrate the proportion of different categories within the dataset, offering insights into the composition and diversity of the sampled construction projects across Saudi Arabia. For instance, Fig. 3 (a) depicts the distribution of Organization Types among the respondents, while Fig. 3 (b) shows the breakdown of Roles in Projects. Similarly, Figs. 3 (c-g) present the distributions for Experience (Years), Project Type, Project Size/Budget, Material Type, and Health and Safety Incidents, respectively. Most respondents were affiliated with contractors and consultants, and project types were predominantly commercial and infrastructure, reflecting the prevailing trends in the Saudi construction sector.

**Fig. 4:**
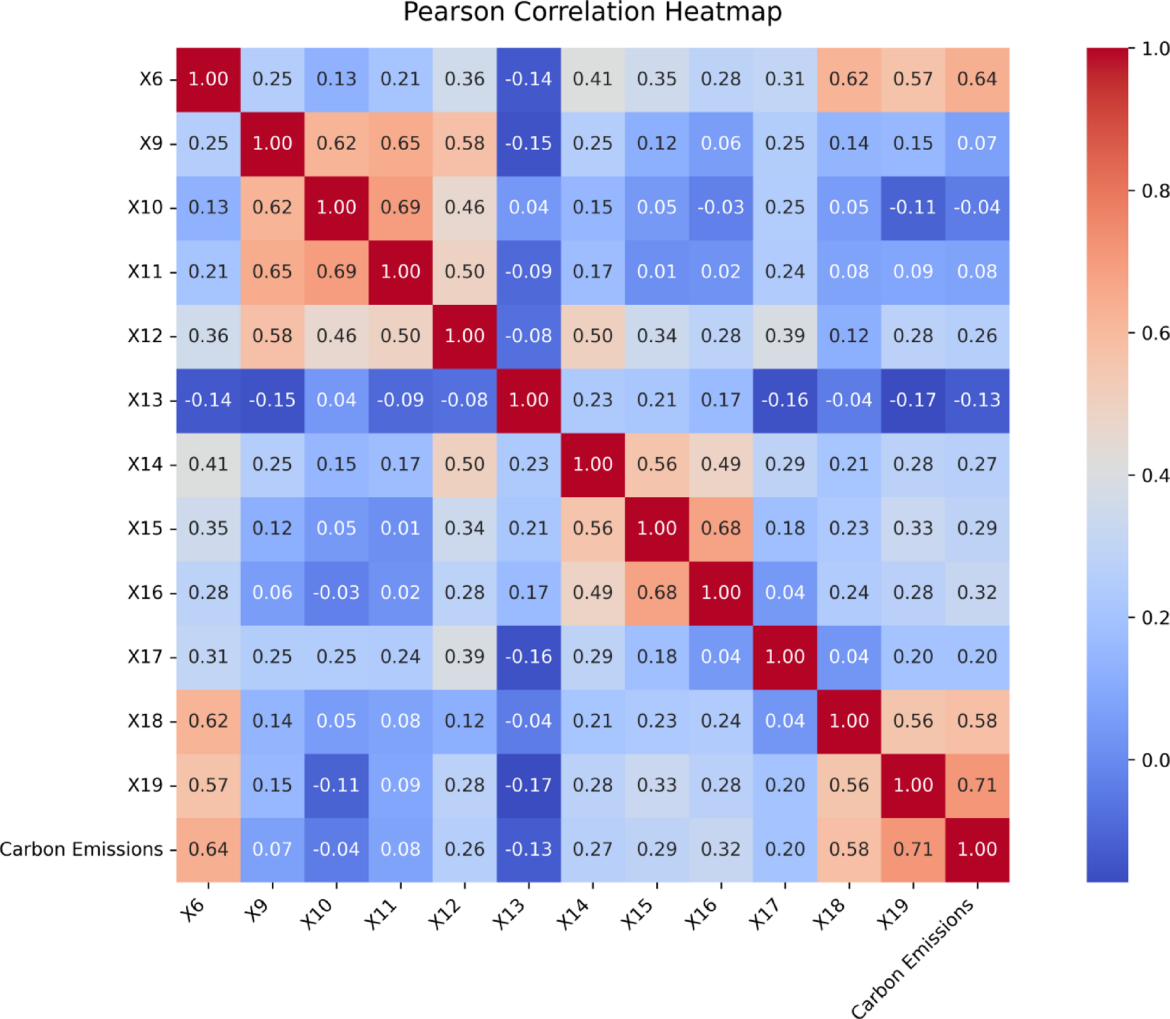
Distribution of respondents’ ratings on the importance of sustainability indicators.

The analysis of numerical variables includes the examination of histograms, especially in relation to the Likert-scale ratings pertaining to distinct indicators of sustainability. These indicators demonstrate participants’ perceptions regarding the consideration of the environment, the economy, and social factors in sustainable construction practices. Histogram also allowed tracking of the quantitative data captured through the five-point Likert scale for other indicators such as Importance of Site Selection, Sustainable Materials, Energy Consumption, Carbon Emissions, Waste Management, Water Usage, Indoor Environmental Quality, Sustainable Project Management, Initial Project Cost, Lifecycle Cost, Return on Investment, Economic Efficiency, Health and Safety, Community Impact, and several more. Health and safety standards as well as community impact also formed part of the ‘social’ indicators of the sustainable development goals (SDGs). The trends are depicted in Fig. 4.

As shown in the histogram (Fig. 4), most respondents rated significant environmental factors such as carbon emissions, energy consumption, and waste management with a 4 or 5, indicating strong recognition of sustainability issues by industry professionals. The economic factors, as well as Life Cycle Cost and Return on Investment, in addition to social factors like Health and Safety, were also considered important for sustainable projects, if not critical. The focus of responses to the upper part of the Likert scale corroborates the importance placed on integrated sustainability performance in the contemporary construction industry.

Moreover, to analyze the features of two critical input parameters, waste generated and energy consumption alongside the primary output variable, violin plots were created (Figs. 3 (h-j)). Violin plots are helpful because they convey distribution and density data using elements from boxplots as well as kernel density estimation, revealing more of the data features. As the plots demonstrate, the value of waste generation (Fig. 3(h)) had an approximate range between 1000 m^3^ to over 160,000 m^3^, signifying significant variability across several projects. Energy consumption exhibited an even greater range with values from 10 million kWh to more than 1.4 billion kWh (Fig. 3(i)), which indicates the significant differences in construction activities’ scale and operational demand. Additionally, the primary output variable carbon emissions (Fig. 3(j)) also exhibited notable dispersion across projects, ranging from 1000 tons to over 450,000 tons. This data demonstrates variability in the construction projects’ performance sustainability benchmarks. Differentiating performance benchmarks suggest the severity and accuracy estimates needed in predictive models for calculating the environmental impact.

Additionally, to assess the relationships among all variables and detect potential multicollinearity, a Pearson correlation heatmap was developed (Fig. 1). The heatmap displays the pairwise correlation coefficients between input features and target outcomes, with color gradients indicating the strength and direction of relationships. While most variables exhibited low-to-moderate correlations, several predictors were found to be conceptually redundant or weakly associated with the target variable. As part of the feature selection process, five variables, Importance of Site Selection, Importance of Carbon Emissions in Assessing Sustainability, Importance of Water Usage, Importance of Sustainable Project Management, and Importance of Community Impact which were initially included in the questionnaire, were removed to avoid redundancy and improve model stability. After this refinement, 19 predictors were retained, and none showed high correlation (|r| > 0.8), confirming the absence of problematic multicollinearity. Understanding these relationships in earlier stages of the analysis is critical when devising a feature selection strategy to improve model stability while avoiding redundant predictors and increasing the resilience of machine learning models^62^.

#### Machine learning techniques

To predict and classify carbon emissions during the construction phase, several supervised ML algorithms were applied in this study. The selected models, SVM, RF, and XGB are well-established for their strong performance on structured tabular data. The algorithms balance accuracy in prediction, computation time, and interpretability. With several models, comparative analysis for both classification and regression can be performed which helps in selecting the best model that represents survey-reported data obtained from construction projects in Saudi Arabia.

##### Support vector machine

**Fig. 5:**
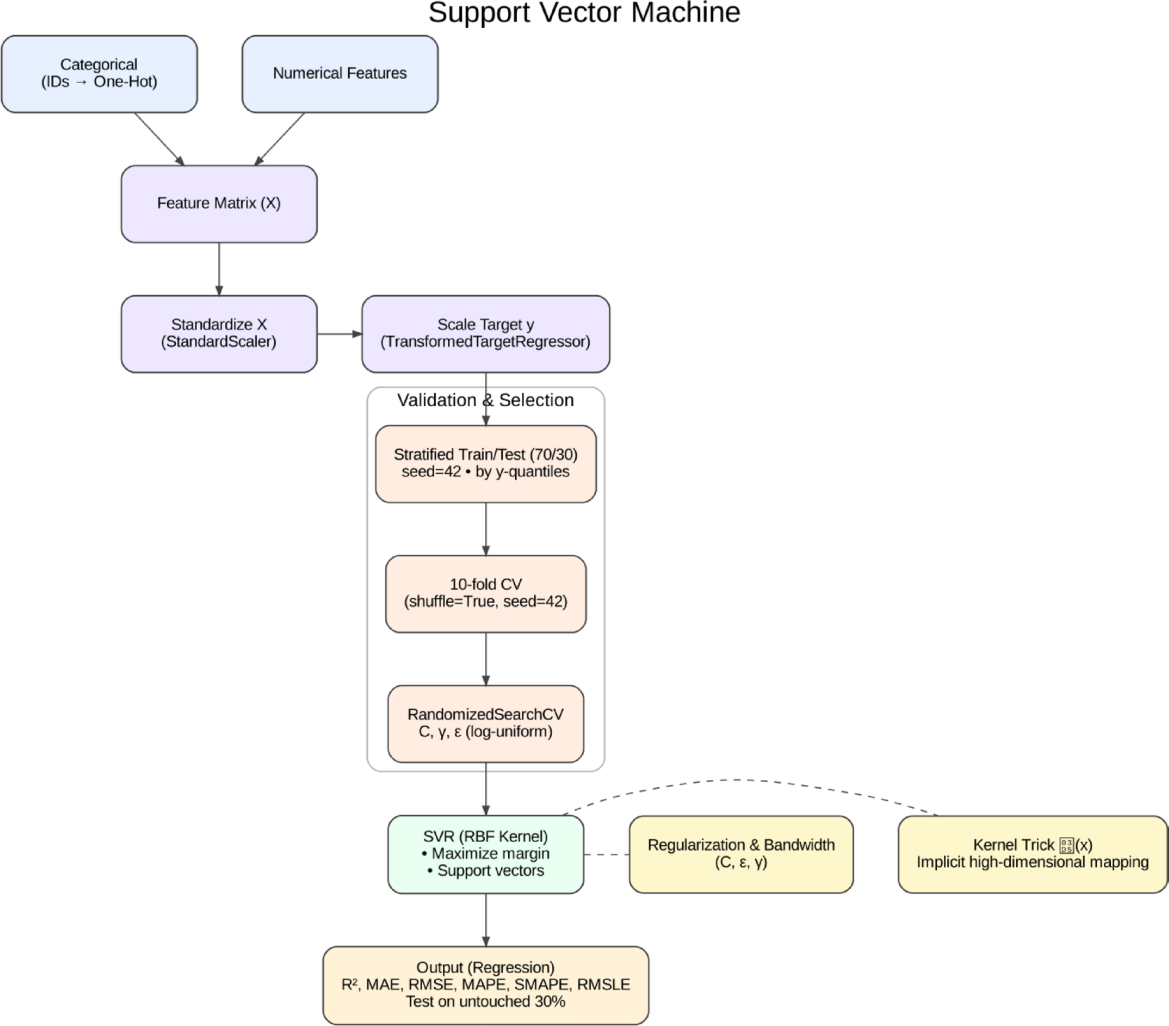
Schematic workflow of the SVM, highlighting feature scaling, cross-validation, hyperparameter tuning, kernel trick, and regression output.

Using kernel functions which transform the input data to higher-dimensional spaces, SVM is an efficient supervised learning algorithm, linear and non-linear relationships. In other words, SVMs perform particularly well in spaces with many dimensions and offer great generalization in cases where the number of observations is small^63–65^. In construction management and sustainability, SVMs have been implemented for estimating project costs, predicting defects, and forecasting energy consumption^34,66^. The structural workflow of the Support Vector Machine (SVM) is illustrated in Fig. 5.

##### Random forest

**Fig. 6:**
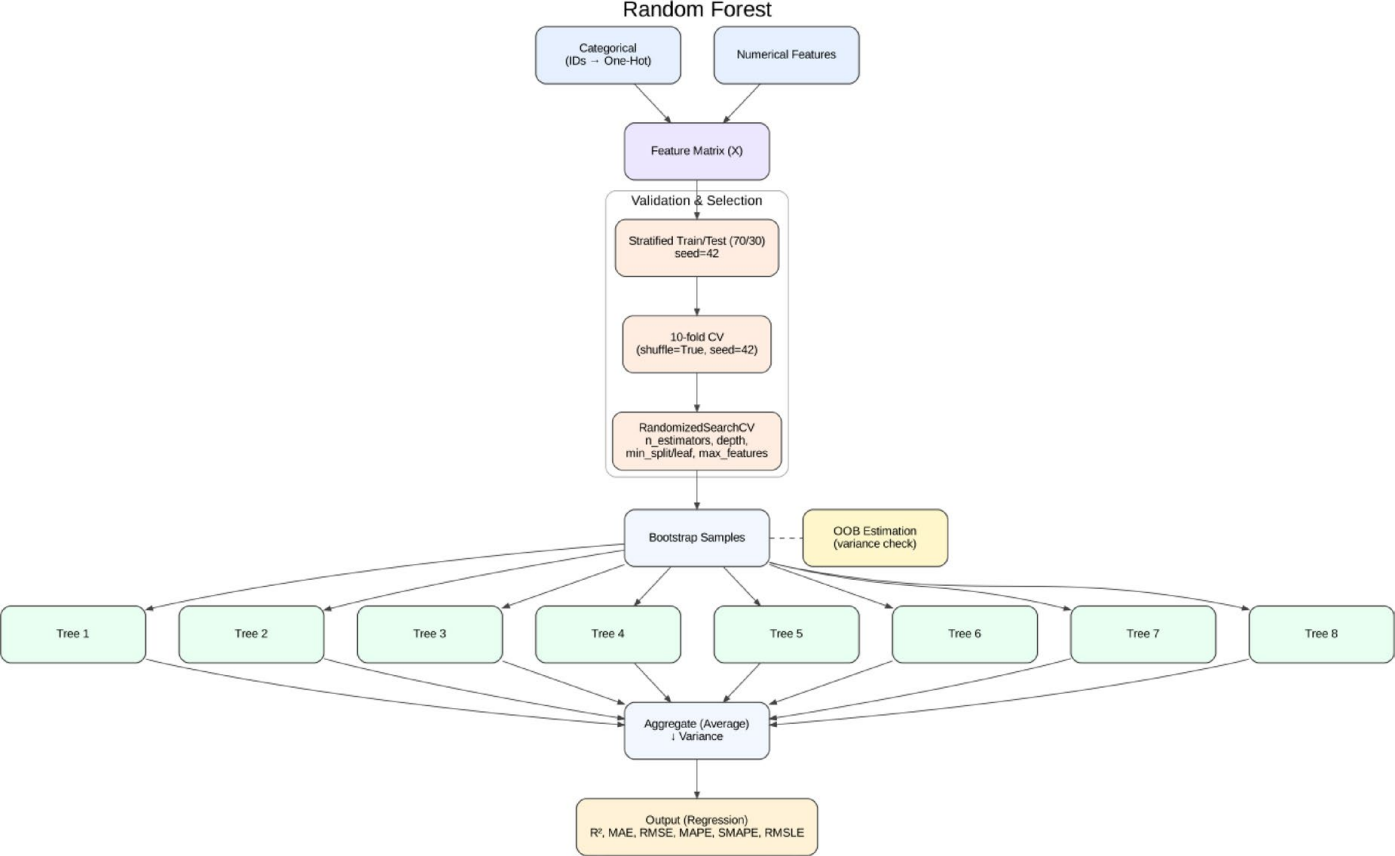
Schematic workflow of the Random Forest model, illustrating feature preprocessing, bootstrap sampling, multiple decision trees, and aggregated regression output.

RF is an ensemble learning model that improves predictive accuracy and robustness by constructing a large number of decision trees and averaging their outputs^26^. Random forests offset the high degree of overfitting associated with individual decision trees through random feature selection and bagging (bootstrap aggregation). For instance, predicting material strength^67^, estimating energy consumption^32^, and supporting the automated making of decisions on designs from a sustainability perspective^68^. The techniques have been extensively used within the construction and sustainability domains. An overview of the Random Forest algorithmic framework is presented in Fig. 6.

##### Extreme gradient boosting

**Fig. 7:**
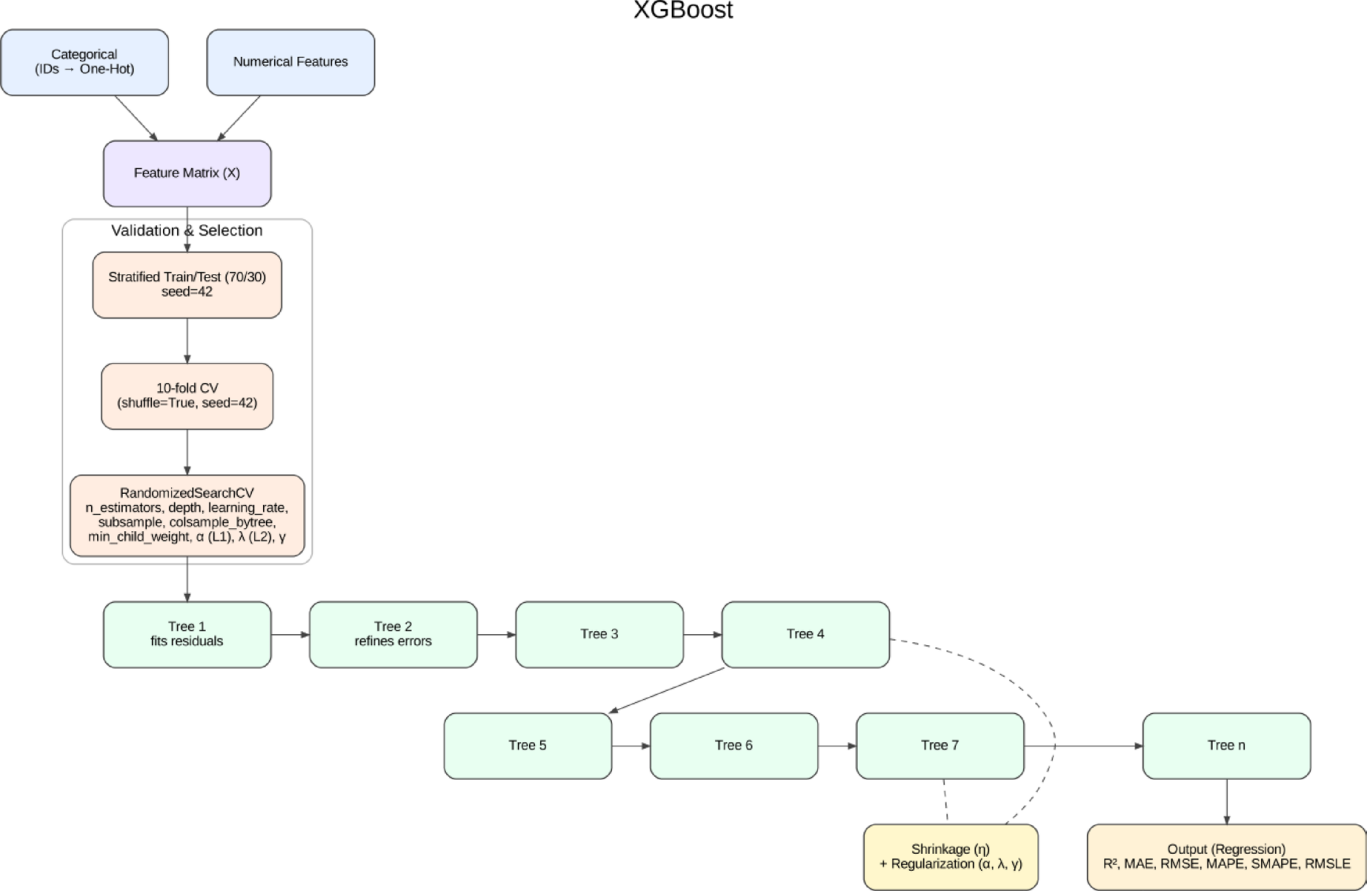
Schematic Workflow of the XGB, showing feature processing, cross-validation, hyperparameter optimization, and boosting sequence.

XGB, a scalable and regularized boosting framework, was also adopted due to its proven track record in machine learning competitions and practical applications^69^. It was also noted that XGB integrates advanced regularization techniques (L1 and L2) which prevent overfitting and parallelized tree construction to further improve training efficiency^70^. Applications of XGB in the construction sector include predicting project delays^71^, constructing risk models in activities^72^ and estimating life-cycle environmental impacts^73^. The schematic representation of the XGB model is shown in Fig. 7.

##### Model comparison and selection rationale

The choice of SVM, RF, and XGB was guided by the characteristics of this study’s data (tabular, mixed-type predictors, *N* = 150 with 19 inputs), the need to model nonlinear relations and feature interactions, and the requirement for transparent interpretation in sustainability assessment. SVM (RBF kernel) is a strong small-sample learner that maximizes margins and captures smooth nonlinearities when variables are properly scaled^63,65^. RF provides variance reduction through bagging, handles heterogenous predictors, is robust to outliers/collinearity, and offers stable baselines on construction datasets^25,26^. XGB (gradient boosting with L1/L2 regularization) was included because boosted trees often outperform bagging on structured tabular data with complex interactions, and they integrate efficiently with TreeSHAP for model interpretation which is critical for actionable sustainability insights^28,69,70^.

Other algorithms were considered considering the same constraints. Linear/GLM baselines were not prioritized because they assume additivity and struggle with the evident nonlinearities in emissions vs. drivers. KNN is distance-based and sensitive to feature scaling/high dimensionality, which is sub-optimal for mixed categorical–numerical inputs. Deep neural networks were not selected as primaries given the modest sample size and heavier tuning demands in construction datasets (though they can excel with large, sensor/BIM-rich corpora)^23,32^. Among boosting variants, LightGBM/CatBoost are credible alternatives, however XGB was chosen for its maturity in the literature, stable performance on small-to-medium tabular data, its advanced regularization, gradient boosting mechanism, and capacity to model intricate feature interactions. Its integration with SHAP also enhances its interpretability, making it highly suitable for sustainability prediction in data-rich environments. Overall, these three models balance generalization, robustness, and interpretability for sustainability assessment in construction. Table 2 further justifying the selection rationale by showing the strengths and comparison between models.

**Table 2:** Model comparison and selection rationale for sustainability prediction^17,25,26,29,63,69,74,75^.

Criteria	SVM	RF	XGB
Handling Nonlinearity	Good with kernels (e.g., RBF), effective in small to medium datasets.	Handles some nonlinearity, but less flexible than XGB or SVM with kernels.	Excellent; captures complex interactions via boosting.
Interpretability	Low; kernel models are less interpretable	Medium; feature importance from tree splits,	Medium; SHAP can enhance interpretability.
Performance on Small Data	Strong generalization with small samples	May overfit unless tuned; needs larger samples.	Performs well with small to large datasets.
Overfitting Risk	Moderate; requires careful parameter tuning.	Low; ensemble nature reduces overfitting.	Lower; includes regularization (L1/L2).
Training Speed	Slow on large datasets.	Faster than SVM; parallel tree training	Fast with optimized libraries.
Suitability for Feature Engineering	Less flexible; sensitive to feature scaling and encoding.	Robust to irrelevant features and noise.	Highly strong; automatically handles missing data and interactions.

#### Data partitioning and model validation

To obtain an unbiased estimate of model generalization and minimize overfitting risk, a nested cross-validation (CV) approach was adopted. The dataset was first divided using an outer 10-fold CV, where each outer fold acted once as a hold-out test set while the remaining nine folds were used for model development. Within each outer training subset, an inner 5-fold CV was conducted to tune model hyperparameters through randomized search. This hierarchical setup ensured that the test data in each outer fold remained completely unseen during model selection, thereby providing a rigorous and unbiased evaluation of model performance.

The inner loop optimized model hyperparameters based on the training data only, using root mean square error (RMSE) and coefficient of determination (R^2^) as the primary performance metrics. Once the optimal hyperparameters were identified, the model was retrained on the entire inner-training subset and evaluated on the corresponding outer-fold test subset. This process was repeated across all ten outer folds, producing distributions of R^2^ and RMSE values that reflected model variability and generalization capability.

A fixed random seed (42) was applied throughout all data splits to ensure reproducibility. This nested CV framework was consistently applied to all machine learning algorithms, including XGB, RF, and SVR. The mean ± standard deviation of the outer-fold R^2^ and RMSE scores were reported as indicators of overall performance stability, while fold-wise results were further analyzed using the Wilcoxon signed-rank test to statistically compare model performances. Hyperparameter tuning ranges and the final selected values for each algorithm are summarized in Table 3.

#### Regression evaluation metrics

The following evaluation metrics were selected to capture both explained variance and error magnitude under skewed, wide-range targets (tons of CO_2_). The study reports R^2^ for explained variance and RMSE/MAE for absolute error magnitude. The Mean Absolute Percentage Error (MAPE) was intentionally excluded due to its instability at small denominators, which leads to disproportionate inflation of percentage errors, particularly relevant here, given emission values spanning approximately 10^3^ to > 4.5 × 10^5^ tons. To address these scaling issues and ensure fairer comparison across emission magnitudes, the study instead reports Symmetric Mean Absolute Percentage Error (SMAPE) and Root Mean Squared Logarithmic Error (RMSLE). These metrics are less sensitive to extreme targets and provide scale-robust, skew-tolerant perspectives that complement MAE and RMSE. For classification (Low/Medium/High), study emphasize macro-averaged Precision/Recall/F1 (treating classes equally under mild imbalance) alongside accuracy and ROC-AUC. This combination follows best practice for tabular, imbalanced, and heteroscedastic outcomes in applied predictive modelling^59^.

- **Coefficient of Determination (R**^**2**^**)** measures the proportion of variance in the dependent variable predictable from the independent variables.

$$\:{R}^{2}=\:1-\frac{{{\Sigma\:}}_{i=1}^{N}{\left({x}_{i}-{\widehat{x}}_{i}\right)}^{2}}{{{\Sigma\:}}_{i=1}^{N}{\left({x}_{i}-\stackrel{-}{x}\right)}^{2}}$$

- **Root Mean Squared Error (RMSE)** quantifies the average magnitude of prediction error.

$$\:RMSE=\:\sqrt{\frac{1}{N}{\sum\:}_{i=1}^{N}{\left({x}_{i}-{\widehat{x}}_{i}\right)}^{2}}$$

- **Mean Absolute Error (MAE)** represents the average absolute difference between actual and predicted values.

$$\:MAE=\:\frac{1}{N}{\sum\:}_{i=1}^{N}\left|{x}_{i}-{\widehat{x}}_{i}\right|$$

- **Symmetric Mean Absolute Percentage Error (SMAPE)** provides a percentage-based error metric that is more robust to zero or near-zero actual values than MAPE. It expresses the average absolute difference between predicted and actual values relative to their mean.

$$\:SMAPE=\:\frac{100\mathrm{\%}}{N}{\sum\:}_{i=1}^{N}\frac{2\bullet\:\left|{x}_{i}-{\widehat{x}}_{i}\right|}{\left|{x}_{i}\right|+\:\left|{\widehat{x}}_{i}\right|}$$

- **Root Mean Squared Logarithmic Error (RMSLE)** measures the square root of the mean squared logarithmic differences between predicted and actual values. It reduces the impact of large outliers and emphasizes relative rather than absolute error, making it particularly useful for datasets with large variance.

$$\:RMSLE=\:\sqrt{\frac{1}{N}{\sum\:}_{i=1}^{N}{(\left(\mathrm{log}1+{x}_{i}\right)-\mathrm{log}(1+{\widehat{x}}_{i}))}^{2}}$$

These choices are particularly relevant in construction sustainability prediction, where emission values can vary widely across project types and scales, and ensuring fair assessment across classes (low/medium/high) is critical for practical decision-making.

#### Classification evaluation metrics

When sustainability outcomes were classified into categories (e.g., Low, Medium, High emissions), classification performance was assessed using:

- **Precision** is the ratio of correctly predicted positive observations to the total predicted positives.
- **Recall (Sensitivity)** is the ratio of correctly predicted positive observations to all actual positives.
- **F1-Score** is the harmonic mean of Precision and Recall, providing a balance between the two.
- **Accuracy** represents the ratio of correctly predicted observations to total observations.
- **ROC-AUC (Receiver Operating Characteristic – Area Under the Curve)** was used to evaluate the trade-off between true positive rate and false positive rate across thresholds for multi-class classification tasks.

## Results and discussions

1.1 Regression Results and Analysis.

The regression tasks aimed to predict carbon emissions based on project characteristics and sustainability factors. Three machine learning models were evaluated: SVM, RF, and XGB. The utilized key hyperparameters which are essential for training and accurate predictions are mentioned in Table 3. Hyperparameters were chosen using randomized search within a nested cross-validation framework (10 outer x 5 inner folds), where the inner loop optimized model parameters and the outer loop provided unbiased generalization estimates. The final selected hyperparameters were then used to refit each model on the full training set before a single evaluation on the untouched 30% test set.

**Table 3:** Hyperparameter settings for applied machine learning Models.

Model	Key Hyperparameters	Values
XGB	Number of Estimators	405
	Maximum Depth	7
	Minimum Child Weight	6
	Learning Rate	0.042
	Sub Sample	0.902
	Col sample by tree	0.874
	Regression Alpha (α)	1.955
	Regression Lambda (λ)	1.320
	Gamma	0.880
	Random State	42
RF	Number of Estimators	301
	Maximum Depth	14
	Maximum Features	0.9
	Minimum Samples per Leaf	1
	Minimum Samples per Split	5
	Boot strap	True
	Random State	42
SVM	Kernel	Radial Basis Function (rbf)
	Regularization Parameter (C)	24.637
	Epsilon	0.3787
	Gamma	0.000184
	Random State	42

**Fig. 8:**
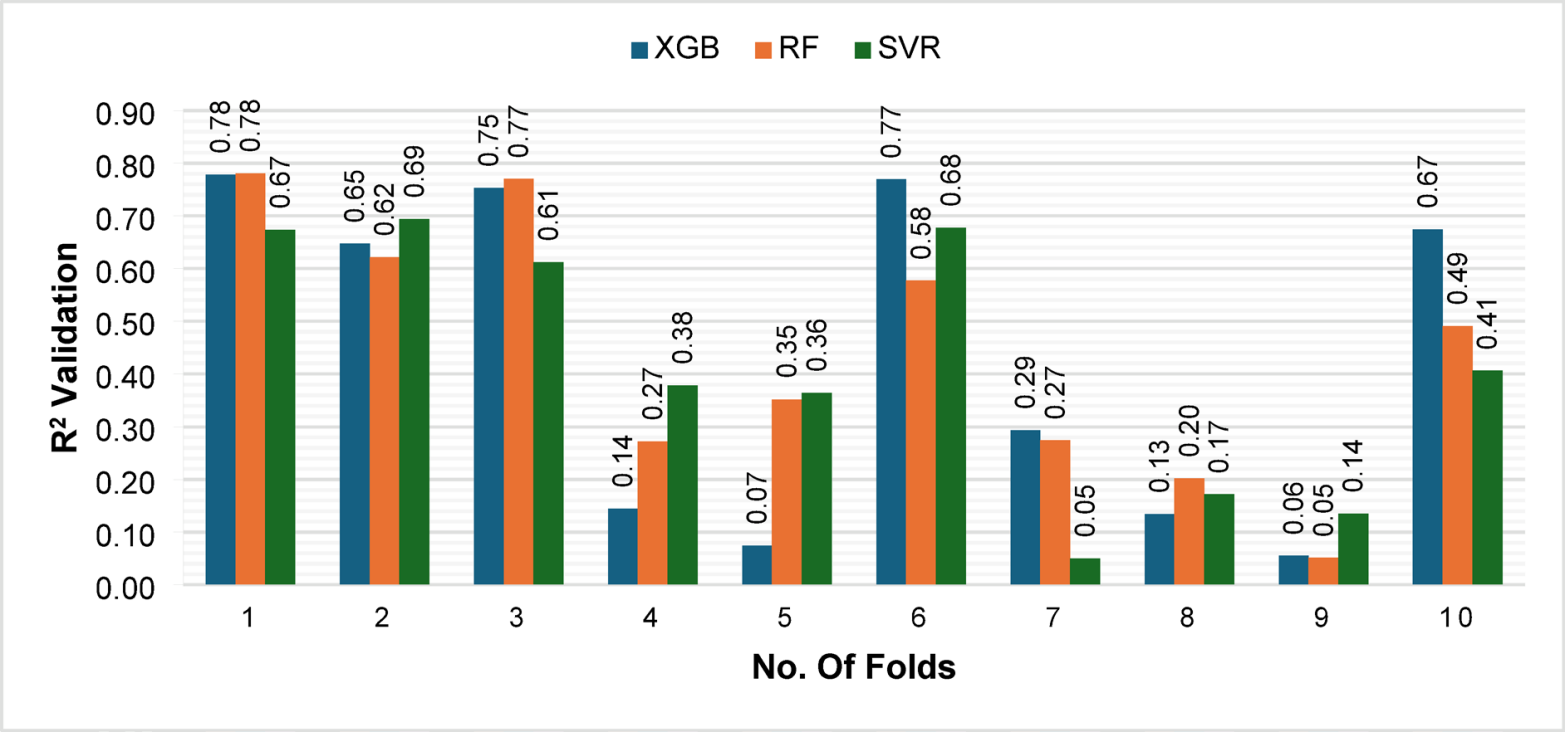
Outer Fold-wise R^2^ values of XGB, RF, and SVM during 10-fold cross-validation.

**Fig. 9:**
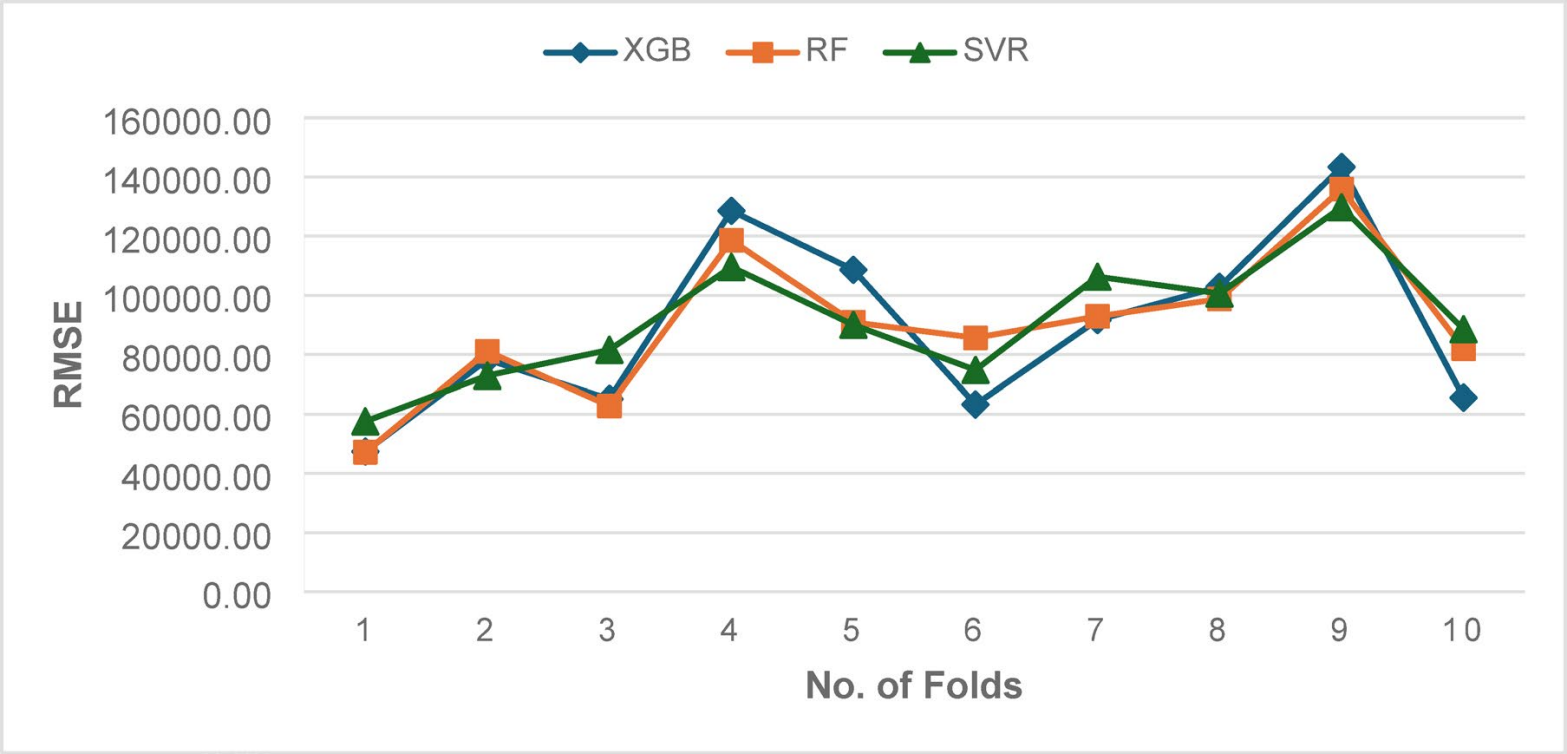
Outer Fold-wise RMSE values of XGB, RF, and SVM during 10-fold cross-validation.

Table 4 summarizes the outer 10-fold CV performance on the training partition (70%) with 95% CI. The mean CV R^2^ values were 0.421 ± 0.332 for XGB, 0.439 ± 0.247 for RF, and 0.416 ± 0.242 for SVM, with corresponding CV RMSE values of 89,471.67 ± 31150.30, 89580.34 ± 25271.81, and 91181.74 ± 21021.62, respectively. The fold-wise trajectories in Fig. 8 (R^2^) and Fig. 9 (RMSE) reveal noticeable variability across folds for all three models, reflecting the wide dispersion of the target variable. Despite this variability, RF consistently achieved higher R^2^ values and lower RMSE than XGB and SVM across most folds, indicating comparatively superior predictive stability.

The relatively large fold-to-fold R^2^ deviations (SD ≈ 0.2–0.3) highlight inherent instability due to heterogeneous target scales and limited sample representativeness per fold. Reporting 95% CIs thus provides a more reliable interpretation of mean performance and uncertainty, effectively quantifying model robustness. These results underscore the necessity of complementing cross-validation summaries with independent test-set evaluation (30% hold-out) to ensure a more generalizable and statistically sound assessment of model performance.

**Table 4:** Outer 10-fold cross-validation summary (training partition, 70%).

Model	CV *R*² (mean ± SD)	95% CI (*R*^2^)	CV RMSE (mean ± SD	95% CI (RMSE)
**XGB**	0.421 ± 0.332	(0.183, 0.660)	89471.67 ± 31150.30	(67188.09, 111755.25)
**RF**	0.439 ± 0.247	(0.262, 0.616)	89580.34 ± 25271.81	(71501.97, 107658.71)
**SVM**	0.416 ± 0.242	(0.243, 0.589)	91181.74 ± 21021.62	(76143.78, 106219.70)

**Fig. 10:**
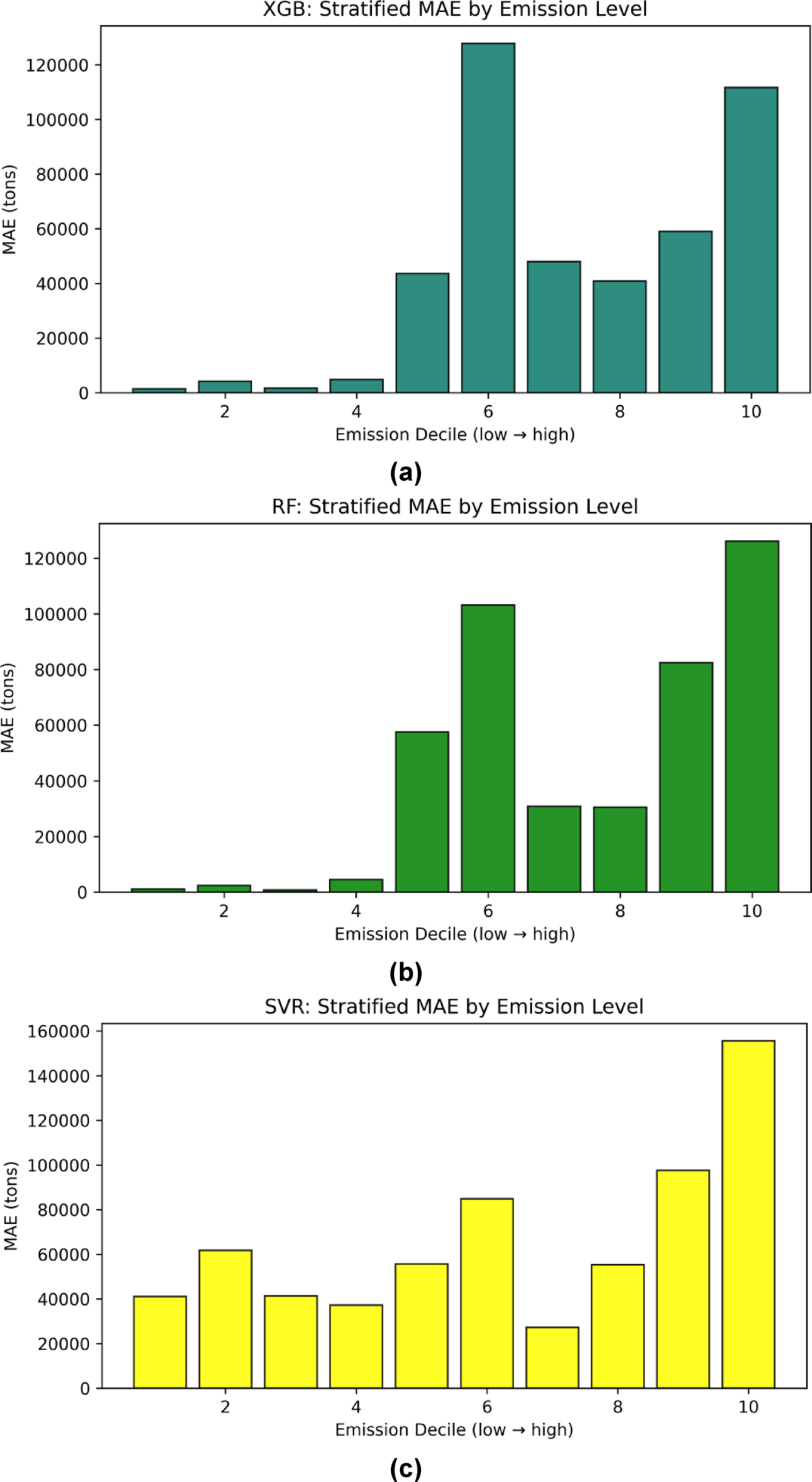
Stratified MAE by emission levels (a) XGB, (b) RF, (c) SVM.

To further diagnose the distribution of error and identify regions of poor generalization, a stratified per-decile MAE analysis was conducted for the independent test set (Fig. 10a-c). The emission range was divided into ten deciles from low to high values, and MAE was computed within each segment. The results reveal that prediction errors were generally lower in the lower-to-mid emission deciles but increased substantially toward the upper range. For instance, the MAE for XGB and RF models remained below 50 000 tons in the lower deciles but exceeded 120 000 tons for the top decile, indicating that both models tend to under-predict high-emission cases. In contrast, SVM exhibited a more uniform but overall higher MAE distribution, with errors peaking at above 150 000 tons in the final decile. This pattern suggests that all models captured low-to-moderate emission levels reasonably well but struggled to generalize for extreme emission values due to the heavy-tailed target distribution. These findings complement the cross-validation outcomes and explain the relatively high MAE and RMSE values reported for the test set, particularly at the upper end of the emission spectrum.

**Fig. 11:**
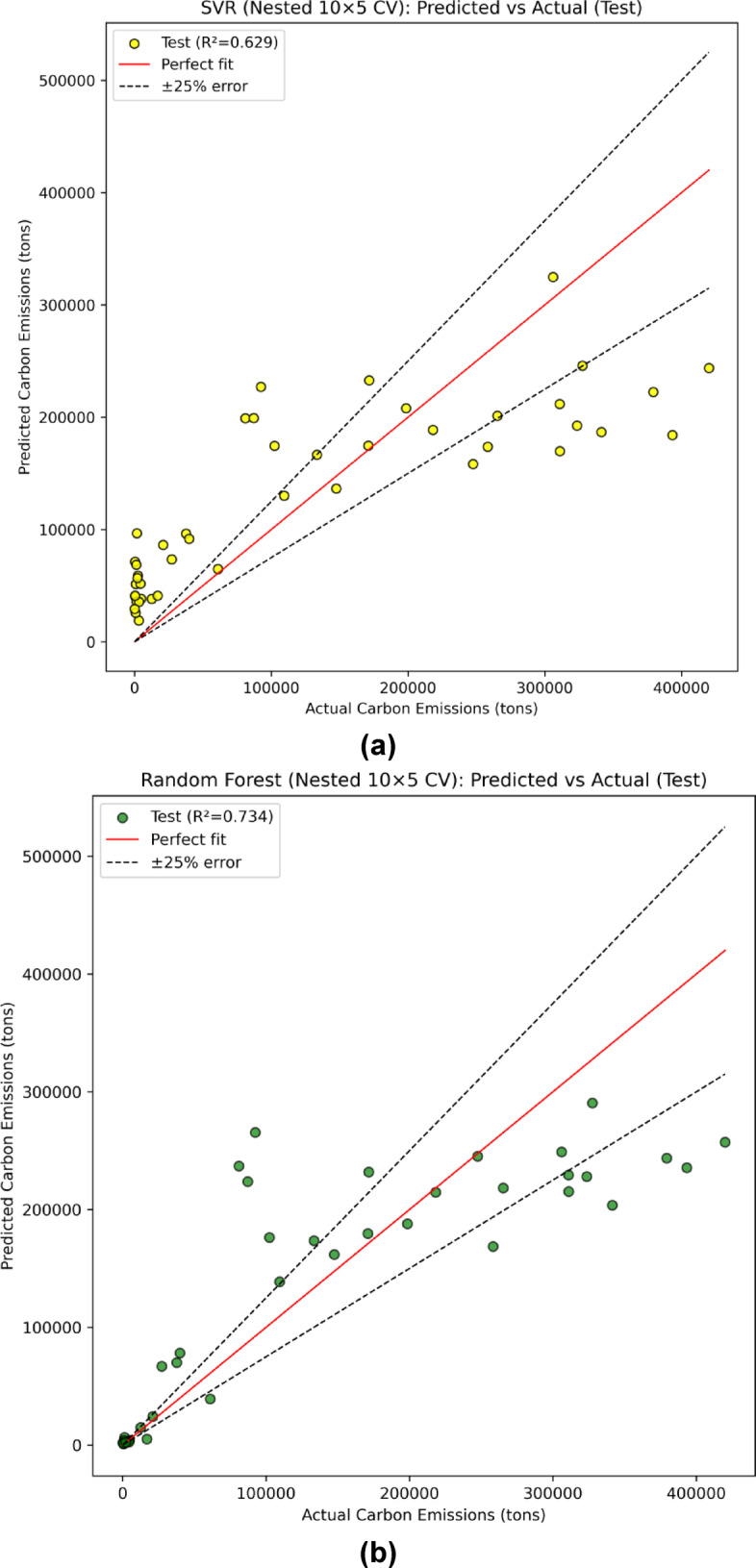

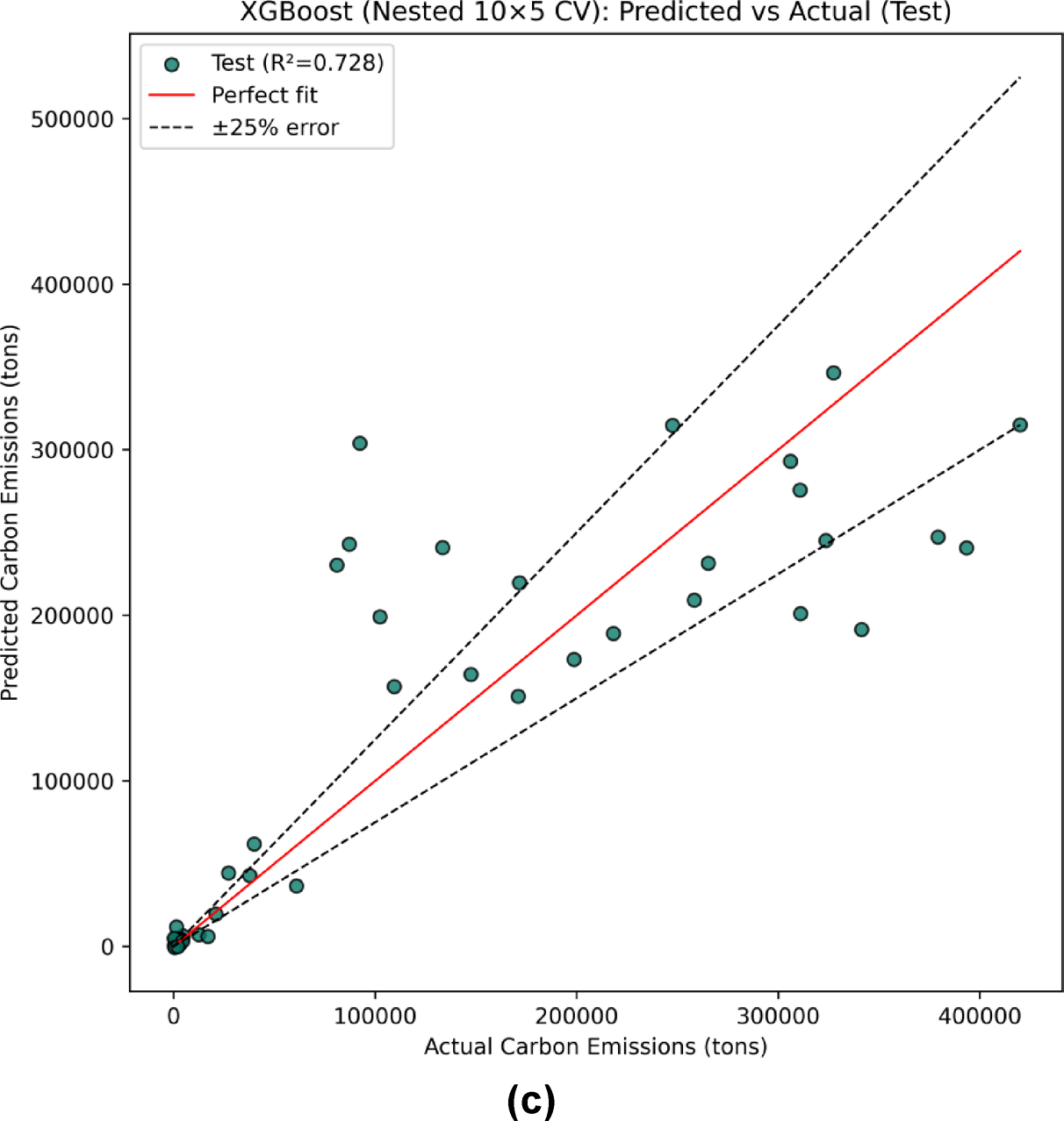
Prediction of carbon emissions on the independent 30% test set using regression models: (a) SVM, (b) RF, (c) XGB.

Figure 11 (a-c) presents the regression curves for the independent 30% hold-out test set following nest 10 × 5-fold cross-validation. The results show that RF achieved the highest predictive fit with R^2^ = 0.734, followed closely by XGB with R^2^ = 0.728, and SVM with R^2^ = 0.629. These outcomes highlight that while RF best explained the variance in the unseen data, XGB also performed competitively and offered more balanced error behavior across evaluation metrics.

**Table 5:** Independent Test-set performance of regression models for carbon emissions prediction.

Evaluation Parameters	SVM	RF	XGB
R^2^	0.629	0.734	0.728
MAE	66228.09	43881.80	43845.04
RMSE	82217.64	69627.39	70377.31
SMAPE	94.02%	48.35%	61.52%
RMSLE	2.15	0.721	1.963

In terms of error metrics (Table 5), the RF model achieved the lowest error values on the independent test set, with an MAE of 43881.80 tons and RMSE of 69627.39 tons, followed closely by XGB (MAE = 43845.04 tons, RMSE = 70377.31 tons). Both ensemble models outperformed SVM (MAE = 66228.09 tons, RMSE = 82217.64 tons), demonstrating their superior capability to capture nonlinear relationships and mitigate overfitting through bootstrap aggregation and boosting mechanisms. The relatively higher errors in SVM reflect its sensitivity to hyperparameter tuning and its limited flexibility in handling datasets characterized by heavy-tailed target distributions such as carbon emissions. To address potential scaling issues in error interpretation, particularly given the extremely wide emission range from approximately 10^3^ to > 4.5 × 10^5^ tons Fig. 3(j), and as also illustrated in the stratified MAE plots (Fig. 10a-c), this study additionally reports SMAPE and RMSLE, which are less sensitive to extreme targets and provide more reliable insight into proportional prediction accuracy. Consistent with MAE and RMSE, RF attained the lowest RMSLE (0.721) and SMAPE (48.35%), confirming its robustness under both absolute and logarithmic error measures.

**Fig. 12:**
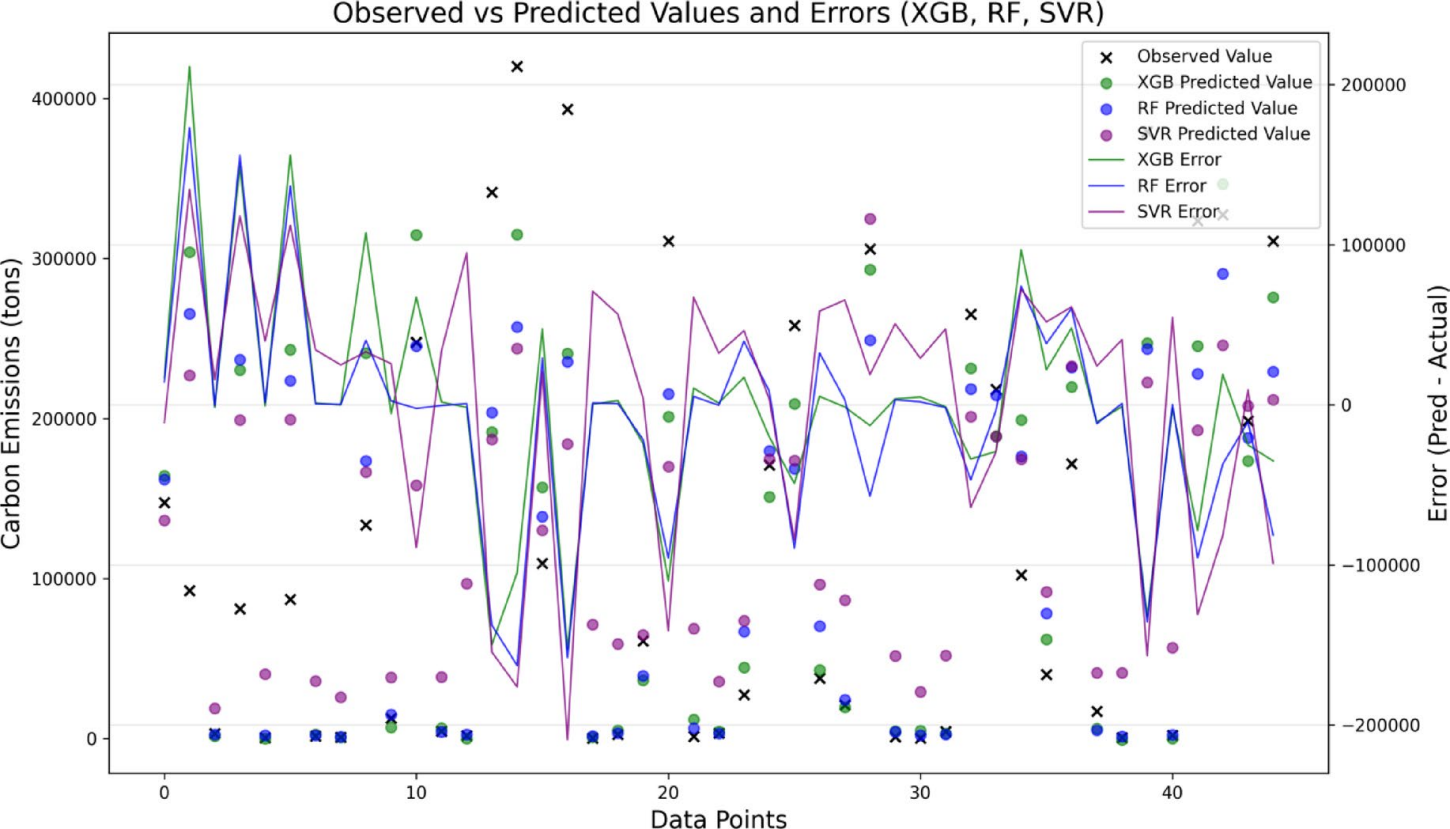
Comparison of observed versus predicted carbon emissions with corresponding error distributions for XGB, RF, and SVM models.

Moreover, Fig. 12 presents a comparative visualization of observed versus predicted emissions and their corresponding residuals for the three models. The plot reveals that RF predictions align more closely with the observed values, reflected by smaller and more stable residuals across most data points. XGB shows comparable performance but with slightly higher fluctuations at extreme emission levels, while SVM demonstrates the highest variance and error magnitudes across the entire range. This observation is consistent with the stratified MAE analysis, which showed that all models experienced rising errors toward higher emission deciles, but RF and XGB maintained better consistency in the mid-to-high range.

To assess whether performance differences among the three regression models were statistically meaningful, a Wilcoxon signed-rank test was conducted on the paired nested cross-validation results as shown in Table 6. The pairwise comparisons between XGB and RF yielded non-significant differences for both RMSE (W = 24, *p* = 0.769) and R^2^ (W = 24, *p* = 0.769), indicating that neither model consistently outperformed the other across folds. Similarly, the comparison between XGB and SVM showed non-significant differences for RMSE (W = 25, *p* = 0.845) and R^2^ (W = 26, *p* = 0.921). Across all cases, p-values exceeded the conventional α = 0.05 threshold, demonstrating that the apparent numerical differences in point estimates are not statistically significant under paired non-parametric testing. This finding is consistent with prior studies applying non-parametric rank-based tests for model comparison in construction materials prediction^76^, where p-values above 0.05 were interpreted as evidence that models share statistically similar predictive behavior rather than one being decisively superior. Under the Wilcoxon signed-rank framework, this corresponds to failing to reject the null hypothesis that the median paired differences between models are zero, meaning the models arise from the same underlying performance distribution. Accordingly, the three regressors can be considered comparably reliable within the data constraints of this study and observed performance differences should be interpreted as practical rather than statistically decisive distinctions.

**Table 6:** Wilcoxon signed-rank test results comparing the paired predictive performance of XGB, RF, and SVM across nested cross-validation folds using RMSE and R^2^.

Pair	Metric	Median Difference	Wilcoxon	‘*p*’ value
XGB vs. RF	RMSE	1272.916	24	0.769
XGB vs. SVM	RMSE	−3958.589	25	0.845
XGB vs. RF	R^2^	−0.0097	24	0.769
XGB vs. SVM	R^2^	0.0274	26	0.921

In summary, the strong performance of RF can be attributed to its ensemble averaging of decorrelated decision trees, which minimizes overfitting and enhances resilience to extreme target values^69,72,77^. In contrast, XGB’s sequential boosting structure enables high accuracy but can occasionally magnify noise in datasets with large target disparities. SVM, while conceptually capable of modeling complex relationships through kernel transformations^19^, often struggles with scale imbalance and hyperparameter sensitivity (e.g., kernel bandwidth, regularization strength), which can lead to unstable predictions when dealing with heavy-tailed distributions^78,79^. Therefore, RF demonstrated the most reliable balance between bias and variance, offering the most robust and interpretable framework for carbon emission estimation.

### Classification results and analysis

For the classification task, carbon-emission outcomes were categorized into three classes, Low, Medium, and High to reflect varying sustainability levels across construction projects. Three well-established machine learning classifiers were employed: SVM, RF, and XGB, representing margin-based learning, bagging ensembles, and boosting ensembles respectively^74,80,81^. The nested cross-validation results (Table 7) showed that RF achieved the highest overall performance, with an accuracy of 0.790 ± 0.094 and a macro F1-score of 0.776 ± 0.101, outperforming SVM (0.753 ± 0.115 accuracy, 0.733 ± 0.136 macro F1) and XGB (0.764 ± 0.136 accuracy, 0.731 ± 0.172 macro F1). AUC values remained consistently strong across models, with macro-average AUCs ranging between 0.885 and 0.922, indicating that despite moderate overlaps in the data, all three classifiers exhibited strong separability between emission classes. These cross-validation trends were reflected in the independent 30% test set (Table 8), where RF again produced the highest test accuracy (78%), followed by XGB (76%) and SVM (73%). Class-wise, SVM demonstrated excellent capability in identifying Low-emission projects with perfect precision (1.00) and high recall (0.93), resulting in an F1-score of 0.97. However, SVM showed more difficulty distinguishing Medium and High classes, yielding F1-scores of only 0.57 and 0.67. RF, in contrast, delivered the most balanced performance across all classes, achieving F1-scores of 0.97 (Low), 0.69 (Medium), and 0.67 (High), with a macro-average F1 of 0.77. XGB performed strongly on medium emissions with perfect recall (1.00) and produced a more even distribution across the three classes (F1 = 0.67 for Low, 0.67 for Medium, 0.62 for High), achieving a macro-average F1 of 0.75.

**Fig. 13:**
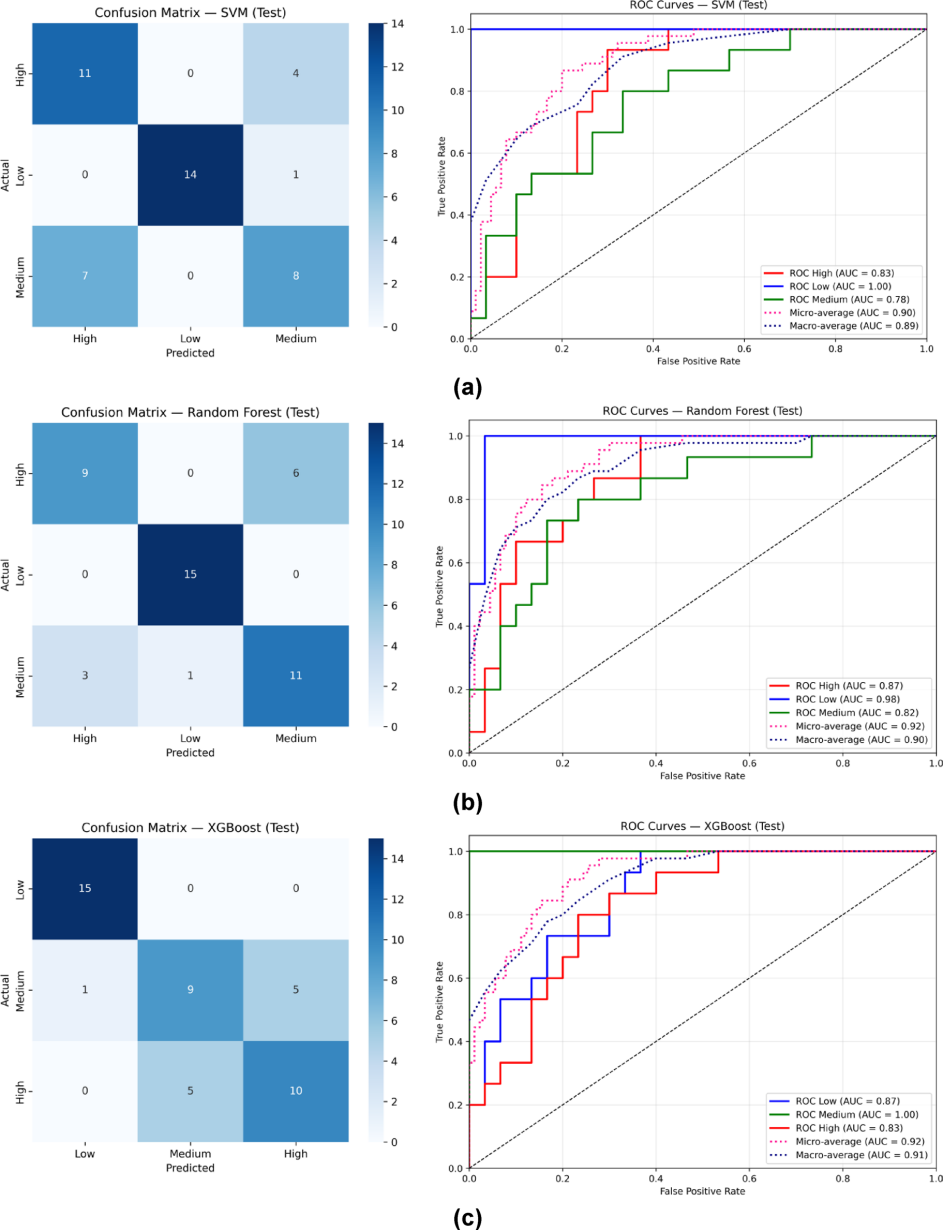
Confusion Matrices and ROC curves (Classification Graphs); (**a**) SVM, (**b**) RF, (**c**) XGB.

The confusion matrices in Fig. 13 (a-c) clarifies these patterns. SVM correctly identified all Low-emission samples but misclassified 7 Medium cases as High and 4 High cases as Medium, showing that the boundary between Medium and High emissions posed a challenge for margin-based separation. RF exhibited fewer confusions, with perfect recall for the Low class (15 correct predictions) and comparatively reduced medium-to-high and high-to-medium misclassification counts (e.g., only 3 Medium misclassified as High and 6 High misclassified as Medium). XGB also achieved perfect recall for the Low class while showing moderate confusion between Medium and High emissions (e.g., 5 Medium misclassified as High and 5 High misclassified as Medium). Across all models, the most prominent misclassification trend involved Medium and High emissions being confused with one another. This is expected, as survey-derived sustainability indicators frequently position projects within overlapping mid-range emission levels, implying limited feature separation between Medium and High categories. In contrast, the Low-emission projects consistently displayed clear feature distinctions, enabling all three classifiers to achieve near-perfect separation for this class.

ROC curves (Fig. 13 (a-c)) further confirmed these findings. SVM achieved AUCs of 1.00 for Low, 0.78 for Medium, and 0.83 for High, with a macro-average of 0.89. RF demonstrated similarly strong performance (Low = 0.98, Medium = 0.82, High = 0.87; macro-average = 0.90). XGB achieved the highest class-specific AUC, obtaining 1.00 for the medium class, and strong values for Low (0.87) and High (0.83), resulting in a macro-average AUC of 0.91. The consistently high AUC values across all models reaffirm that, although accuracy and F1 values were moderated by overlapping Medium-High class characteristics, the underlying class boundaries remained generally identifiable.

Overall, RF emerged as the most strong and stable classifier for this dataset, benefiting from variance reduction and strong generalization. XGB excelled in refining decision boundaries, particularly for medium emissions, achieving the highest ROC separability. SVM maintained exceptional precision for Low-emission projects but struggled with Medium-High distinctions due to the inherent overlap in survey-based sustainability indicators^63,75^. Across all models, the recurring confusion between Medium and High emissions indicates that future work may benefit from enhanced feature engineering or additional survey variables to improve class separability in mid-emission ranges.

**Table 7:** Nested cross-validation performance of the SVM, RF and XGB classifiers across sustainability-related carbon-emission classes.

Model	Metric	Accuracy	F1 Macro	AUC Macro	AUC Micro
**SVM**	Mean ± SD	0.753 ± 0.115	0.733 ± 0.136	0.908 ± 0.066	0.918 ± 0.049
	95% CI	(0.670, 0.836)	(0.635, 0.830)	(0.860, 0.955)	(0.883, 0.954)
**RF**	Mean ± SD	0.790 ± 0.094	0.776 ± 0.101	0.922 ± 0.051	0.940 ± 0.031
	95% CI	(0.723, 0.858)	(0.703, 0.849)	(0.885, 0.958)	(0.918, 0.963)
**XGB**	Mean ± SD	0.764 ± 0.136	0.731 ± 0.172	0.899 ± 0.067	0.931 ± 0.044
	95% CI	(0.666, 0.862)	(0.607, 0.854)	(0.850, 0.947)	(0.899, 0.963)

**Table 8:** Class-wise performance evaluation of the SVM, RF, and XGB classifiers on the independent 30% test set.

Model	Class	Precision	Recall	F1-Score	Accuracy (%)
SVM	Low	1.00	0.93	0.97	73
	Medium	0.62	0.53	0.57	
	High	0.61	0.73	0.67	
	Macro Avg	0.74	0.73	0.73	
RF	Low	0.94	1.00	0.97	78
	Medium	0.65	0.73	0.69	
	High	0.75	0.60	0.67	
	Macro Avg	0.78	0.78	0.77	
XGB	Low	0.67	0.67	0.67	76
	Medium	0.94	1.00	0.97	
	High	0.64	0.60	0.62	
	Macro Avg	0.75	0.76	0.75	

### SHAP and PDP interpretation analysis

**Fig. 14:**
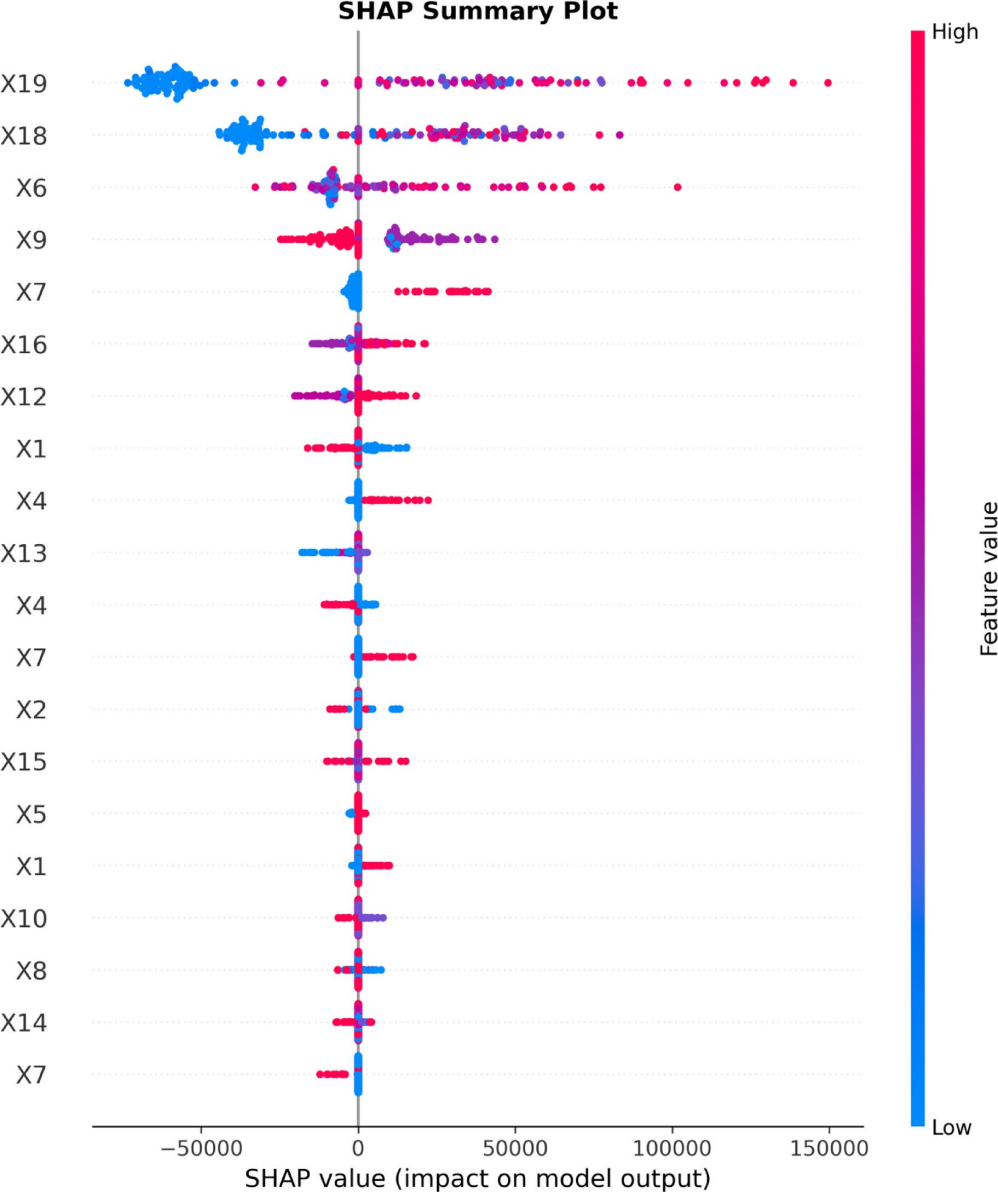
SHAP Interpretation analysis (Summary plot).

**Fig. 15:**
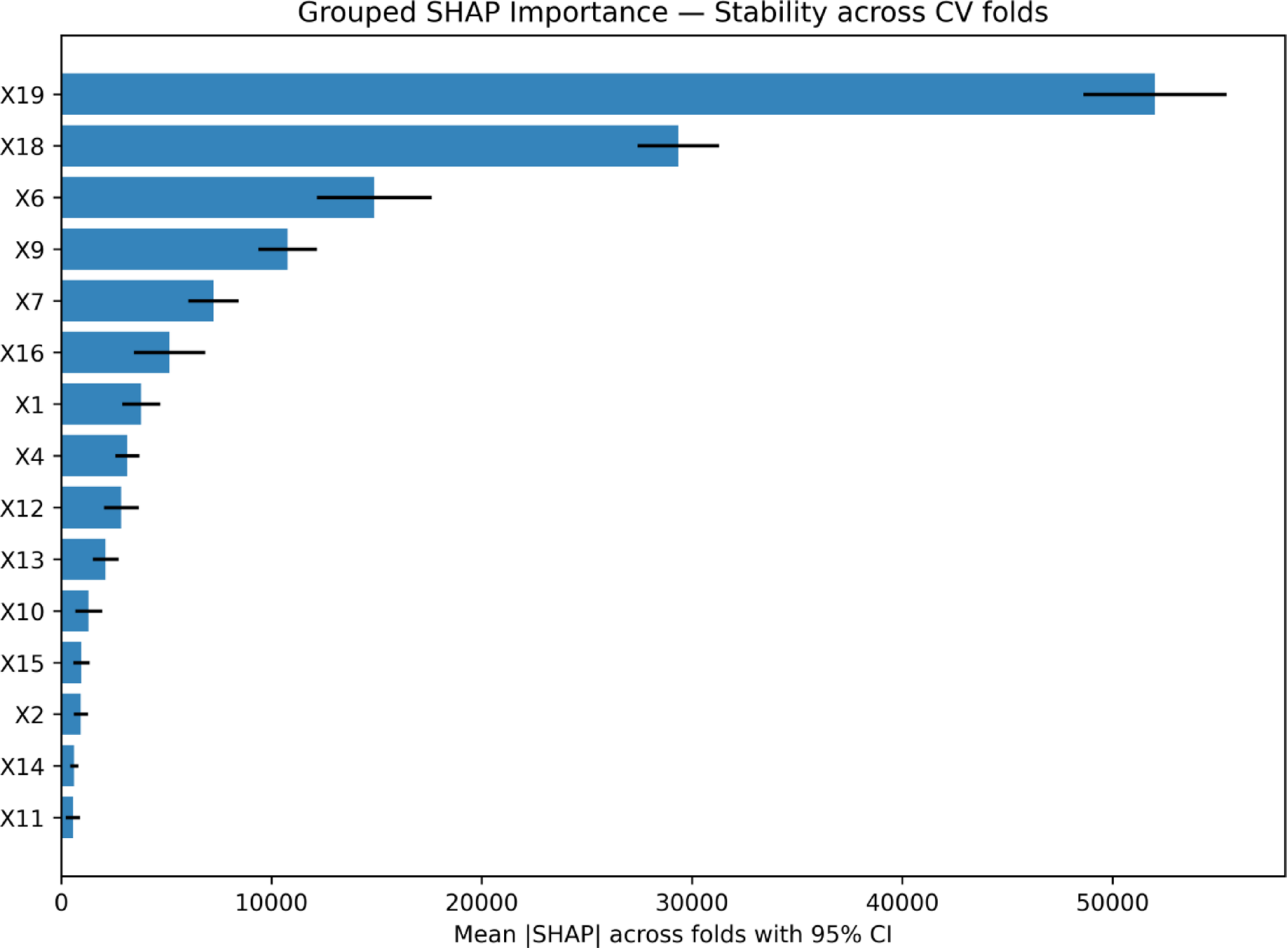
SHAP Feature important analysis.

To enhance interpretability and understand the underlying drivers of carbon-emission predictions, a comprehensive SHAP (SHapley Additive exPlanations) analysis was performed using the XGB model, supported by multi-fold stability checks, dependence plots, interaction effects, and partial dependence curves^82^. The SHAP summary plot (Fig. 14) consistently identified Waste Generated (X19), Energy Consumption (X18), Project Duration (X6), and Importance of Sustainable Materials (X9) as the most influential predictors of project-level carbon emissions. High values of X19 and X18 were strongly associated with large positive SHAP contributions, confirming that projects with excessive material wastage or substantial energy use tended to produce disproportionately higher emissions. This ranking was further validated through grouped SHAP importance analysis (Fig. 15), where the mean absolute SHAP values across cross-validation folds showed X19 (≈ 54,000), X18 (≈ 34,000), and X6 (≈ 18,000) as the top contributors. Importantly, the feature-ranking stability analysis demonstrated strong consistency across folds, with an average Spearman rank correlation of 0.803, confirming that the SHAP explanations were reliable and not artifacts of individual data splits.

**Fig. 16:**
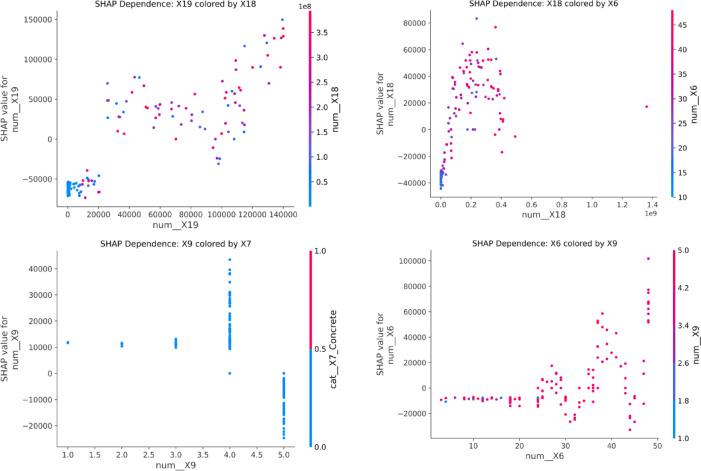
SHAP Dependence plots.

The SHAP dependence plots (Fig. 16) provided deeper insights into marginal feature effects and interactions. X19 exhibited a clear monotonic upward trend, where increases in waste volume from 10,000 m^3^ to over 130,000 m^3^ were associated with rapid increases in SHAP values exceeding 150,000, indicating a dominant influence on emissions. For X18, the dependence relationship showed a steep rise in SHAP contributions within the lower-to-mid energy consumption range, followed by stabilization beyond approximately 1.0 × 10^9^ kWh, suggesting diminishing marginal impact at extremely high levels of energy use. Project Duration (X6) demonstrated a nonlinear effect, where durations below 15 months contributed minimally, but projects extending beyond 35–40 months produced substantially higher SHAP values, aligning with literature emphasizing that longer operational phases intensify fuel use, site activities, and resource consumption. Interestingly, the dependence plots revealed notable interaction effects: X19’s influence increased more sharply when X18 was simultaneously high, as reflected in the color gradients, indicating compounding impacts of waste generation and energy intensity. Likewise, X6 showed interaction-driven inflections when conditioned on X9 (importance of sustainable materials), implying that projects emphasizing sustainable material selection tended to mitigate some duration-related emissions^83,84^.

**Fig. 17:**
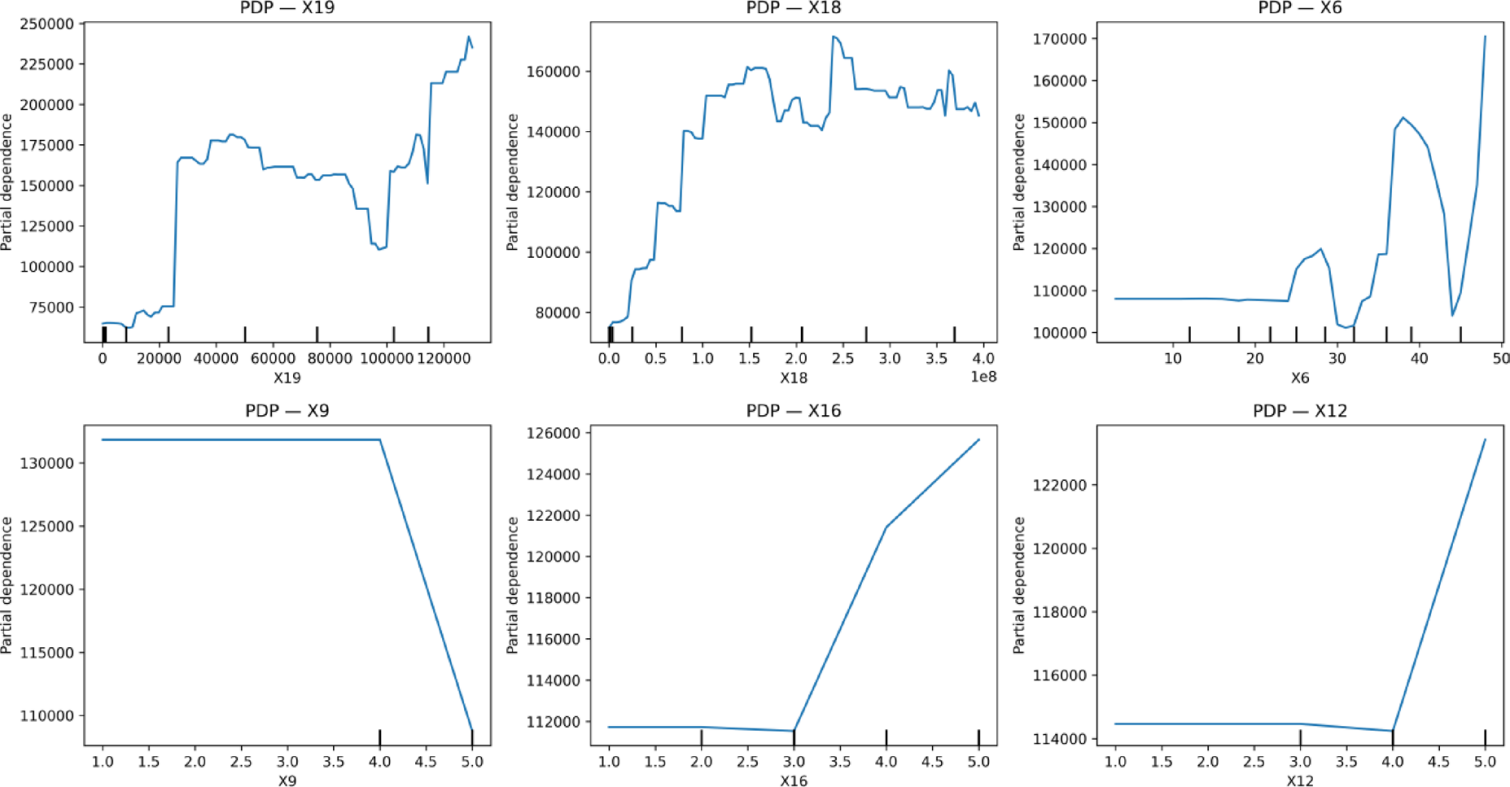
Partial Dependence Plots.

To complement SHAP analysis, Partial Dependence Plots (PDPs) were generated for the top predictors to validate overall model behavior (Fig. 17). The PDP for X19 displayed a strong globally increasing trend, confirming the monotonic relationship observed in SHAP. The PDP for X18 exhibited a plateauing pattern beyond the mid-range values, consistent with SHAP’s saturation effect. Similarly, PDPs for X6, X16 (Economic Efficiency), and X12 (Indoor Environmental Quality Importance) corroborated the SHAP-based findings by showing nonlinear but meaningful effects on predicted emissions. Together, these visualizations strengthened the causal interpretability of the model by demonstrating that the learned relationships were smooth, stable, and aligned with domain expectations.

Across the dataset, the magnitude of SHAP values reflected substantial heterogeneity in project characteristics: Waste Generated ranged from less than 5,000 m^3^ to over 160,000 m^3^, while Energy Consumption spanned from approximately 1 × 10^7^ to more than 1.4 × 10^9^ kWh. This broad variability partially explains why model errors were more pronounced in low-emission projects and why rare extreme-emission projects contributed heavily to the model’s learning dynamics. Nevertheless, the integration of SHAP-based explanations with cross-validation stability, interaction inspection, and PDP verification significantly enhances transparency and interpretive trust in the model. These insights allow practitioners to quantify the marginal emission contributions of individual project attributes and identify the specific conditions such as excessive waste, energy-intensive operations, or prolonged construction cycles under which emissions escalate most sharply.

The findings have immediate application in project workflows. During early design and tendering, the XGB-based predictor can provide “what-if” estimates of project CO_2_ given design choices (e.g., material mix, expected duration, equipment intensity), allowing teams to compare alternatives and select lower-emission options before execution. Contractors can embed the model in weekly dashboards to benchmark sites, with SHAP explanations highlighting actionable hotspots (e.g., high waste generation, excessive energy consumption, prolonged duration) and triggering targeted interventions (improved waste segregation, optimized equipment schedules, resequencing). Consultants can integrate predictions as a decision-support layer alongside LCA to prioritize scenarios for detailed assessment, and clients/policymakers can incorporate forecasted emissions into pre-qualification and performance clauses (e.g., bonus0malus tied to predicted/realized emissions). The model can also complement existing rating systems (e.g., LEED, BREEAM) by supplying early-stage quantitative evidence for credits related to construction-phase impacts, without replacing formal LCA requirements. These uses translate the model’s accuracy and explainability into concrete planning, monitoring, and procurement decisions.

### Policy recommendations

The findings of this study underscore the growing importance of integrating advanced predictive analytics into sustainability assessment practices during the construction phase. Despite the availability of sustainability frameworks, proactive management of environmental performance remains limited, particularly during active project execution. By leveraging machine learning models such as XGB, construction managers and policymakers can move from reactive monitoring to anticipatory decision-making, identifying sustainability risks before they materialize. Therefore, informed by the results of this study, several policy recommendations are proposed to bridge the gap between predictive insights and actionable sustainability practices in the Saudi Arabian construction sector.

- **Integration of AI and ML Tools**:

Government agencies and large construction firms should integrate machine learning models like XGB into sustainability assessment protocols to enable early detection of high-risk projects and corrective interventions.

- **Data-Driven Decision-Making**:

Sustainability reporting requirements should be updated to mandate the collection and analysis of quantitative indicators such as waste generation, energy consumption, and project duration. Policies encouraging digital data acquisition (IoT sensors, smart meters) will enhance prediction accuracy.

- **Incentives for Sustainable Construction**:

Policymakers should provide incentives (tax breaks, certification advantages) to projects that demonstrate proactive management of critical sustainability factors identified through ML-driven insights.

- **Mandatory Environmental Audits**:

Environmental audits based on predictive models could be institutionalized at different project stages (planning, mid-construction, completion) to ensure adherence to sustainability targets.

- **Capacity Building and Training**:

Professional training programs focusing on AI/ML for sustainability assessment should be introduced for engineers, project managers, and policymakers to bridge the digital skills gap in the Saudi construction sector.

## Limitations and future perspectives

### Research limitations

This study relies on cross-sectional, survey-derived project data from six major Saudi cities. Although instrument validation and pilot testing reduced ambiguity, self-reporting and recall can introduce measurement error in numerical outcomes (carbon emissions, energy use, waste), and the geographic focus may limit generalizability to other regions, delivery methods, or regulatory contexts. The sample size (*N* = 150) relative to the number of inputs (19) and the skewed, wide range of target values can inflate percentage-based error metrics despite reporting SMAPE and RMSLE; residual heteroscedasticity may persist. While SHAP improves transparency of the XGB model, the attributions are associative rather than causal and should not be interpreted as evidence of intervention effects. External validation on independent cohorts and prospective, operational deployment were beyond this study’s scope.

### Future perspectives

Based on the current study’s important insights into the predictive modeling of sustainability outcomes during construction, several future research directions are provided below:

- Expand the dataset to include larger, multi-regional samples from diverse project types (residential, commercial, infrastructure) to improve generalizability and reliability.
- Combining tree-based models with deep learning architectures (e.g., Deep Neural Networks, LSTM) could capture more complex temporal or sequential patterns in construction activities once time-indexed data (e.g., phase-level emissions, equipment-hours, or activity logs) becomes available.
- Real-time or near-real-time prediction frameworks, fed by on-site IoT devices and Building Information Modeling (BIM) systems, could revolutionize proactive environmental management during project execution.
- Advanced explainability techniques (e.g., Deep SHAP, Counterfactual Explanations) to uncover deeper insights into the causal relationships influencing sustainability outcomes.
- Integration of ML-based predictive tools into regulatory frameworks to enable automated early warning systems for non-compliance with green building standards.
- Explore the use of explicit interaction terms and automated feature selection techniques (e.g., recursive elimination, mutual information) to improve interpretability and efficiency, particularly for models sensitive to feature configurations.

Advancing research along these directions will strengthen the role of AI-driven decision support in achieving Saudi Arabia’s Vision 2030 sustainability targets and the broader global SDGs.

## Conclusions

This study presents a comprehensive framework for predicting carbon emissions during the construction stage of projects using advanced machine learning techniques. Based on a carefully developed and circulated survey targeting major Saudi Arabian cities, the research systematically applied SVM, RF, and XGB algorithms to model sustainability outcomes. Additionally, SHAP analysis was employed to enhance model interpretability and identify the most influential factors contributing to environmental impacts.

- Incorporating nested 10 × 5-fold cross-validation on the 70% training partition, RF demonstrated the strongest generalization performance, achieving the highest mean CV R^2^ (0.439 ± 0.247) and the lowest CV-RMSE (89,580 ± 25,272). XGB (CV R^2^ = 0.421 ± 0.332) and SVM (0.416 ± 0.242) followed closely, both exhibiting moderate fold-to-fold variability.
- On the independent 30% test set, RF again achieved the highest variance explanation (R^2^ = 0.734), outperforming XGB (0.728) and SVM (0.629). RF also delivered the lowest RMSE (69,627 tons) and the most favorable percentage-based accuracy (SMAPE = 48.35%). XGB and SVM showed competitive performance but with higher sensitivity to extreme emission values.
- In absolute error terms, XGB and RF recorded the lowest MAE values (43,845 tons and 43,882 tons respectively), while SVM exhibited higher absolute and percentage errors. RF achieved the lowest RMSLE (0.721), indicating stronger robustness in capturing multiplicative/severity-scaled error behavior.
- In the classification task, carbon-emission magnitudes were categorized into Low, Medium, and High classes. Nested cross-validation revealed RF as the top-performing classifier, achieving the highest accuracy (0.790 ± 0.094) and macro F1-score (0.776 ± 0.101), followed by XGB and SVM. All models produced strong macro-AUC values (0.885–0.922), confirming solid class separability across folds.
- On the independent 30% test set, RF again attained the highest accuracy (78%), followed by XGB (76%) and SVM (73%). RF also demonstrated the most balanced class-wise performance, whereas SVM excelled mainly in identifying Low-emission projects.
- ROC curve analysis validated the practical discrimination capability of all models, with RF and XGB showing the strongest macro-average AUC values (≈ 0.90–0.91), indicating reliable detection of sustainability-critical emission categories.
- SHAP analysis confirmed that Waste Generated (X19), Energy Consumption (X18), and Project Duration (X6) were the most influential drivers of carbon emissions. These features consistently produced the largest absolute SHAP values across folds.
- The dependence plots highlighted clear positive, nonlinear relationships between these predictors and emission levels such as steep SHAP increases at high waste or energy levels offering actionable insights for project managers seeking to minimize carbon intensities in construction operations.

Taken together, the cross-validated results, independent test-set evaluation, and interpretability analyses suggest an effective modelling strategy: use RF as the primary model due to its stability, balanced performance, and superior error control; employ XGB as a competitive alternative for high-variance prediction settings; and use SVM where strong separability of low-emission cases is desired. These insights support early sustainability risk detection and informed decision-making during planning and execution phases of construction projects.

## Data Availability

The de-identified dataset used in this study is available from the corresponding author, Dr. Saleh Alsulamy (s.alsulamy@kku.edu.sa), upon reasonable request. Public sharing is restricted to protect participant confidentiality.
